# Remarkable hexafunctional anion receptor with operational urea-based *inner cleft* and thiourea-based *outer cleft*: Novel design with high-efficiency for sulfate binding

**DOI:** 10.1038/s41598-017-05831-x

**Published:** 2017-07-20

**Authors:** Maryam Emami Khansari, Ali Mirchi, Avijit Pramanik, Corey R. Johnson, Jerzy Leszczynski, Md. Alamgir Hossain

**Affiliations:** Department of Chemistry and Biochemistry, Jackson State University, Jackson, MS 39217 USA

## Abstract

The recognition of anions by designed receptors has attracted much attention in recent days. In particular, the selective binding of sulfate with artificial receptors is important because of its relevance to many biological and environmental applications. However, the development of organized molecular receptors with high-efficiency for sulfate binding still remains a significant challenge. We report a novel *para*-phenylene-bridged hexafunctional tripodal receptor that contains a urea-based *inner cleft* and a thiourea-based *outer cleft*, providing perfect sites for step-wise binding of two anions within a single cavity. The new receptor was synthesized in a three-step process, and was investigated for its anion binding properties by ^1^H NMR titrations, 2D NOESY experiments and computational studies. As indicated by solution binding studies, the receptor selectively binds sulfate over other oxoanions, forming a 1:2 stoichiometric complex that is stabilized *via* strong H-bonding interactions. High-level DFT calculations reveal that the receptor, owing to the enhanced H-bonding ability of thiourea groups, initially encapsulates one sulfate in its thiourea-based *outer cleft*, followed by a second encapsulation in its urea-based *inner cleft*. Such a functionalized receptor with the unique combination of *urea*-*based cleft* and *thiourea*-*based cleft* in a single receptor has not been reported previously.

## Introduction

Anion binding and sensing with designed abiotic receptors has become an active area of current research^1–6^. Molecular clefts with organized functional groups in defined geometries have recently gained significant attention in anion coordination chemistry^7–10^. Among the various anions, sulfate is particularly important because of its relevance to biological and environmental implications with respect to nuclear waste management^11^, biosynthesis^12^, and protein binding^13^. Thus, there is a growing interest in developing suitable molecular receptors that can strongly and selectively bind sulfate anions^14^. The use of urea groups appended to the tripodal framework in anion binding is well documented, showing complementary binding with sulfate^15–29^. For instance, tren-based urea receptors substituted with *m*-cyanophenyl^15^, *p*-cyanophenyl^16^, *m*-nitrophenyl^17^, and 3-pyridyl^18^ were shown to bind sulfate by hydrogen bonding interactions. Although receptors incorporating urea functional groups have been shown as useful binding motifs for anions through NH···O interactions, it has been reported that the incorporation of thiourea groups to synthetic receptors leads to an enhanced acidity of NH^25^, thereby providing strong affinity for anions^30–38^. Furthermore, recent reports have established the link between the anion binding and certain catalytic reactions, especially for thiourea-based anion receptors^39–41^. For example, Jacobsen *et al*. have reported a thiourea-based compound that, upon the binding of a fluoride ion, can catalyze the acylation of silyl ketene acetals with acyl fluorides^39^.

It has been shown that the free energies of association for host-guest interactions are dependent on the number of rotatable bonds formed by the hosts and guests^42^; therefore, the increased number of binding sites within a single host designed from structural manipulations could lead to the enhancement of its binding ability and selectivity for a specific guest. Recently, Wu *et al*. have reported tripodal-based hexaurea receptors containing *ortho*-phenylene-bridged *bis*urea moieties that encapsulated sulfate through H-bonds, forming 1:1 complexes^43, 44^. In the pursuit of achieving the higher order of binding sites, we synthesized a pentaflouro-substituted hexaurea receptor that formed an encapsulated complex with a carbonate ion^45^.

**Figure 1:**
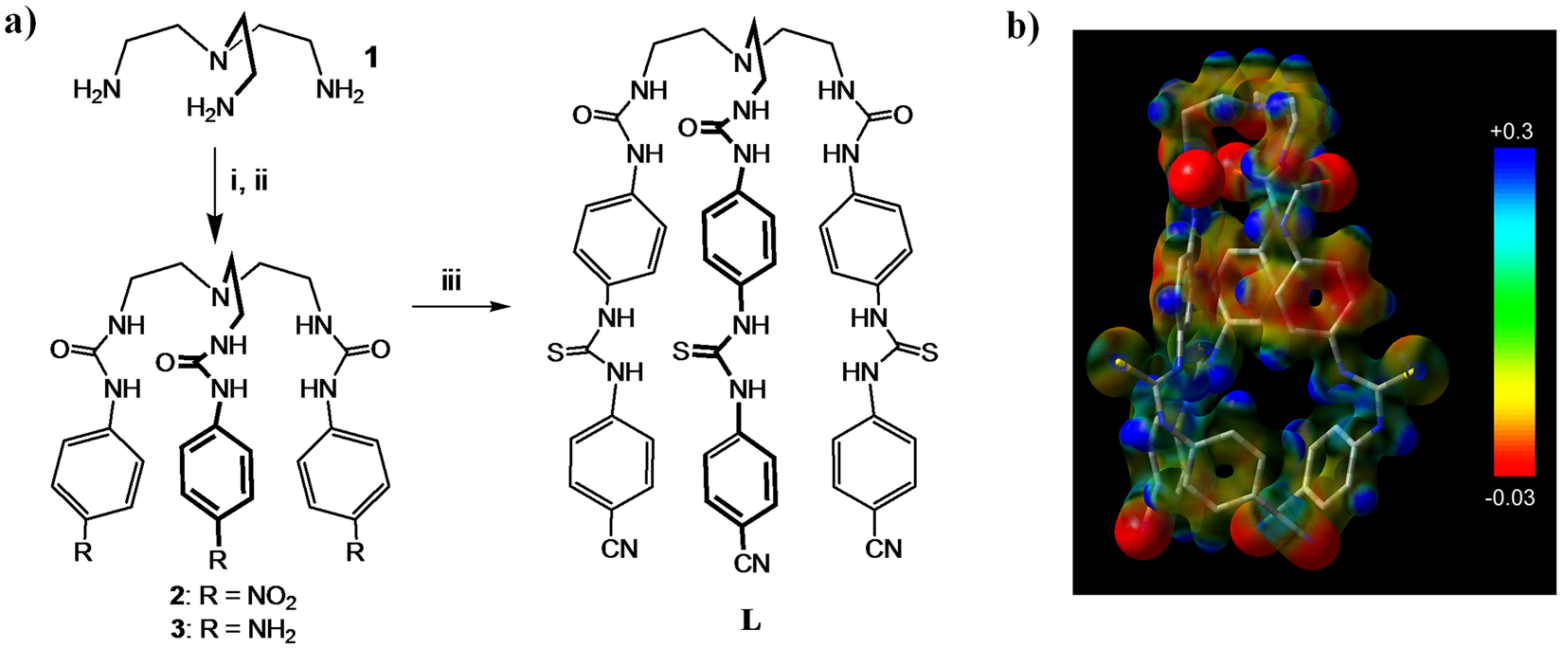
(**a**) Synthetic scheme for **L** ((i) *p*-nitrophenyl isocyanate, (ii) Hydrazine hydrate and Pd/C (10%), and (iii) *p*-cyanophenyl isothiocyanate), and (**b**) electrostatic potential map for **L**, calculated at the M06-2X/6-31G(d,p) level of theory (red = negative potential, and blue = positive potential).

Herein, we report a novel *para*-phenylene-bridged hexafunctional mixed urea/thiourea receptor **L** that contains one *inner cleft* with three urea groups and one *outer cleft* with three thiourea groups. We hypothesized that such an organization with two operational clefts linking through rotatable spacers in a single molecule could provide perfect sites for hosting two anions, each within one cleft. Because of the enhanced acidity of the thiourea groups, the receptor would possibly show preference to bind the first anion at the *outer cleft* (instead of *inner cleft*). This assumption was further supported by the electrostatic potential surfaces of **L** calculated at the M06-2X/6-31G(d,p) level of theory, showing more positive potential on the *outer cleft* than the *inner cleft* (Fig. 1). Such an assembled, exceptional anion receptor with an elite blend of *urea*-*based cleft* and *thiourea*-*based cleft* in a single tripodal receptor has not been reported previously.

## Results and Discussion

As demonstrated by the electrostatic potential map as well as by the optimized structures (discussed later), the receptor adopts into a *C*_*3*_ symmetric cone shape, owing to the presence of three identical (*para*-phenylene-bridged) arms linked to the tertiary amine (Fig. 1). A strong electrostatic positive potential is created within both the *inner* and *outer clefts* due to the urea and thiourea moieties, potentially making the molecule a ditopic receptor for anions. Through the analysis of ^1^H NMR binding isotherms and NMR NOESY experiments, we have shown that the receptor binds sulfate selectively over other oxoanions, forming a 1:2 stoichiometric complex. High level DFT calculations further support that the receptor efficiently encapsulates two sulfate ions in its *inner cleft* and *outer cleft*.

### Synthesis

The new hexafunctional mixed urea/thiourea receptor **L** was prepared by three-step synthetic strategy (Fig. 1a), with about 40% overall yield. *Tris*(2-aminoethyl) amine (tren) (**1**) and *p*-nitrophenyl isocyanate were reacted to give the nitro-functionalized *tris*-urea **2** which was reduced with hydrazine hydrate and Pd/C (10%) to produce the amino-functionalized *tris*-urea **3**. The final coupling was achieved by reacting **3** with *p*-cyanophenyl isothiocyanate to form the *p*-phenylene bridged hexafunctional mixed urea/thiourea **L**. The receptor is stable under normal conditions and soluble in DMSO, but hardly soluble in water and other common organic solvents. Attempts to isolate X-ray quality crystals of **L** with anions were unsuccessful.

### NMR studies

**Figure 2:**
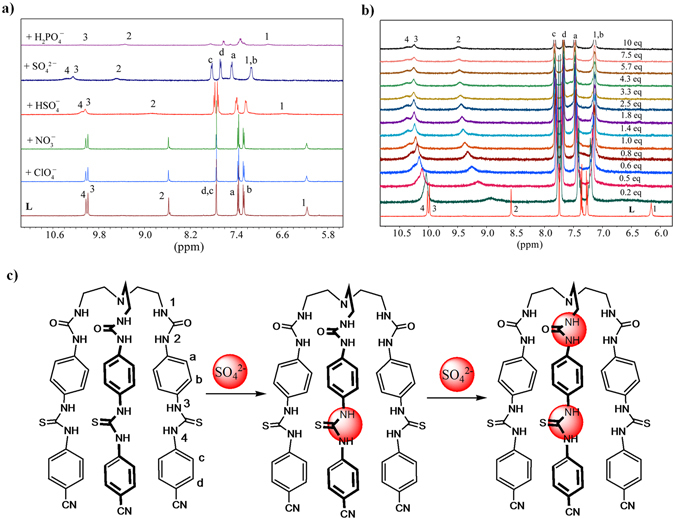
(**a**) Partial ^1^H NMR spectra of **L** (2 mM) in the presence of 5 equivalents of different anions in DMSO-*d*_*6*_; (**b**) partial ^1^HNMR titration of **L** showing changes in the NH chemical shifts of **L** (2 mM) with an increasing amount of SO_4_^2−^ (20 mM) in DMSO-*d*_*6*_. (H1 = CH_2_N*H*CO, H2 = CON*H*Ar, H3 = ArN*H*CS, H4 = CSN*H*Ar); and (**c**) proposed binding mechanism of **L** with SO_4_^2−^.

The binding properties of **L** towards various oxoanions (SO_4_^2−^, HSO_4_^−^, H_2_PO_4_^−^, ClO_4_^−^ and NO_3_^−^), which were added as their tetrabutylammonium (TBA) salts, were investigated in DMSO-*d*_*6*_ by using proton NMR titration techniques at room temperature. The free receptor shows four NH resonances at 6.16 (NH1), 8.58 (NH2), 9.99 (NH3) and 10.02 (NH4) ppm in its NMR spectrum: two for ureas (NH1 and NH2), and the other two for thioureas (NH3 and NH4), indicating the *C*_*3*_ conformation of **L** (The NH signals were assigned by NOESY NMR spectroscopy, see below. See Fig. 2c for the numbering of the NH protons). Figure 2a shows the ^1^H NMR spectra of the free **L** and its mixture with 5 equivalents of different anions. After the addition of SO_4_^2−^ to **L**, the NH resonances significantly shifted downfield showing ΔNH2 = 0.89, ΔNH3 = 0.27 and ΔNH4 = 0.35, while H1 signal overlapped with one aromatic proton at 7.13 ppm (ΔNH1 = 0.97 ppm), suggesting the interactions of **L** with sulfate anions. In addition to the shifting of NH signals, the aromatic signals also shifted. In particular the upfield shift of peripheral signals (Hd and Hb) on *p*-cyanophenyl and *p*-phenylene groups were observed, which may be due to a shielding effect induced by the encapsulated sulfate inside the *outer cleft* ^33, 43^. Notably, the shift difference of NH resonances of urea groups is much larger than that of thiourea groups, suggesting a possible cavity strain due to the encapsulation of sulfate anion into the inner cavity. The addition of HSO_4_^−^ to **L** induced small but considerable changes in the chemical shifts of both urea and thiourea groups, showing ΔNH1 = 0.37, ΔNH2 = 0.28, ΔNH3 = 0.06 and ΔNH4 = 0.09 ppm. The larger shift change (ΔNH) in the respective NH signal due to the addition of SO_4_^2−^ than that of HSO_4_^−^ indicates stronger interactions of SO_4_^2−^ as it contains two charges. The addition of H_2_PO_4_^−^ to **L** resulted in downfield shifts of NH1 and NH2 signals, while both NH3 and NH4 signals were broadened as observed previously for related ligands^43, 44^. In contrast, the addition of ClO_4_^−^ or NO_3_^−^ to **L** did not show any noticeable change in the shifts of NH or aromatic protons (see the Supporting Information, Figs S12 and S13), thus indicating weaker interactions between the perchlorate or nitrate anions and the receptor.

**Figure 3:**
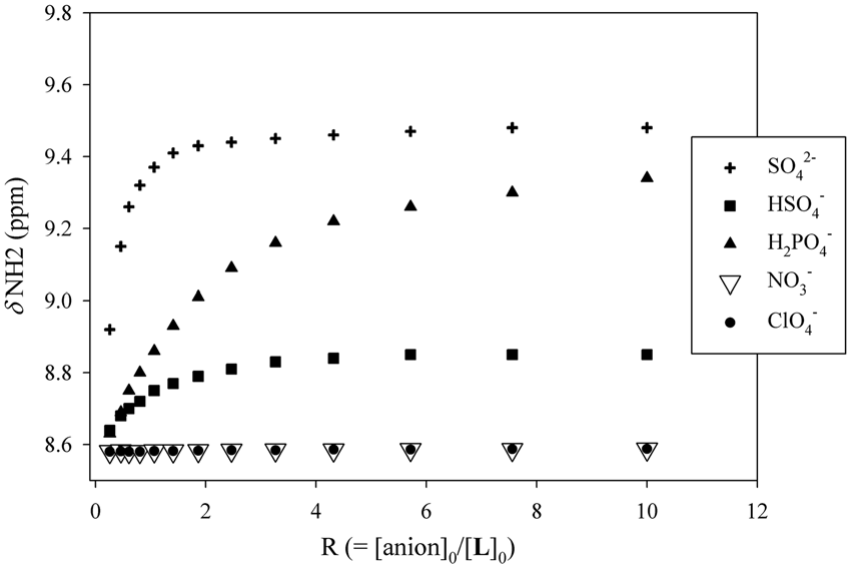
^1^H NMR titration curves of **L** (2 mM) with an increasing amount of various oxoanions (R = [anion]_0_/[**L**]_0_) in DMSO-*d*_*6*_.

Figure 2b shows the stacking of ^1^H NMR spectra of **L** with varying amount of SO_4_^2−^ (0 to 10 eq.) in DMSO-*d*_*6*_, exhibiting gradual downfield shifts of NH signals. The shift changes for NH resonances (Fig. 3), however, were not consistent with a purely 1:1 binding model as commonly observed for related molecules^33^. Therefore, they were analyzed with a 1:2 (**L**:sulfate) binding model using the EQNMR program^46^, displaying the binding constants (in log *K*) of 3.06(2) and 2.56(4) for **L** + SO_4_^2−^ = [**L**(SO_4_)]^2−^ and [**L**(SO_4_)]^2−^ + SO_4_^2−^ = [**L**(SO_4_)_2_]^4−^, respectively. These results clearly indicate the stepwise binding of two sulfates, one with the *inner cleft* and other with the *outer cleft*. Gunnlaugsson *et al*. reported *ortho*-, *meta*-, and *para*-phenylene bridged acyclic urea-amide based receptors, demonstrating that an anion recognition at the first binding moiety may lead to a “positive allosteric effect” for the second functionality toward anions, thus promoting the formation of a 1:2 complex^47^. A similar effect was recently described by Wu *et al*. for a ferrocenyl-functionalized hexaurea receptor that contains two urea groups separated by a *meta*-phenylene group, showing both 1:1 and 1:2 complexes with sulfate anions, as supported by ^1^H NMR and theoretical calculations^44^. In an earlier report, we also observed that a *para*-xylene bridged hexaprotonated azamacrocycle was capable of hosting two chlorides at its two binding moieties *via* trigonal recognition of two clefts^48^. In the present work, the receptor **L** featuring two clefts with different functionalities (urea and thiourea) can readily host two tetrahedral sulfate anions in its two clefts. Owing to an enhanced binding ability as well as the structural complementarity of thiourea functionalities, it is suggested that the first binding occurs at the *outer cleft* followed by the second binding at the *inner cleft*. This is further supported by optimizing the geometries and calculating the respective binding energies using high-level density functional theory (discussed later). As shown in Fig. 2b, the larger change in chemical shifts are observed within 0 to 1 equivalents of SO_4_^2−^, implying a 1:1 complex, while the formation of the 1:2 species is dominant after one equivalent of the added anion. The stepwise binding constants of **L** for SO_4_^2−^ have been shown as log *K*_*1*_ = 3.07(3) and log *K*_*2*_ = 2.56(4) for the first and second sulfate, respectively. Since the first binding constant is higher than the second binding constant, this binding process can be considered as “non-cooperative”^49^. The titrations of **L** with HSO_4_^−^ or H_2_PO_4_^−^ also suggest the stepwise formation of both 1:1 and 1:2 complexes, and the calculated binding constants are provided in Table 1. The higher binding constant for the first step as compared to that for the second step for each complex implies that the outer cavity is the preferential binding site for sulfate, presumably the enhanced acidity of thioureas. Further, the overall binding trend in the order of SO_4_^2−^ > HSO_4_^−^ > H_2_PO_4_^−^ > ClO_4_^−^ or NO_3_^−^, suggests that the receptor can selectively bind sulfate over other anions studied.

**Table 1:** Binding constants of **L** for anions (A) in DMSO-*d*_*6*_^*a*^.

*Anions*	Log *K*_*1*_ (or Log *β*_*1*_)	Log *K*_*2*_	Log *β*_*2*_ (*β*_*2*_ = *K*_*1*_ *K*_*2*_)
SO_4_^2−^	3.07(3)	2.56(4)	5.63(4)
HSO_4_^−^	2.41(5)	1.65(5)	4.06(5)
H_2_PO_4_^−^	2.06(5)	1.75(3)	3.81(5)
ClO_4_^−^	<1	<1	<1
NO_3_^−^	<1	<1	<1

^a^The binding constants were determined using a 1:2 binding model for the following reaction: ${\bf{L}}+{\rm{A}}\mathop{\rightleftharpoons }\limits^{{K}_{1}}[{\bf{L}}{\rm{A}}]\underset{A}{\overset{{K}_{2}}{\rightleftharpoons }}[{\bf{L}}{{\rm{A}}}_{2}]$.

**Figure 4:**
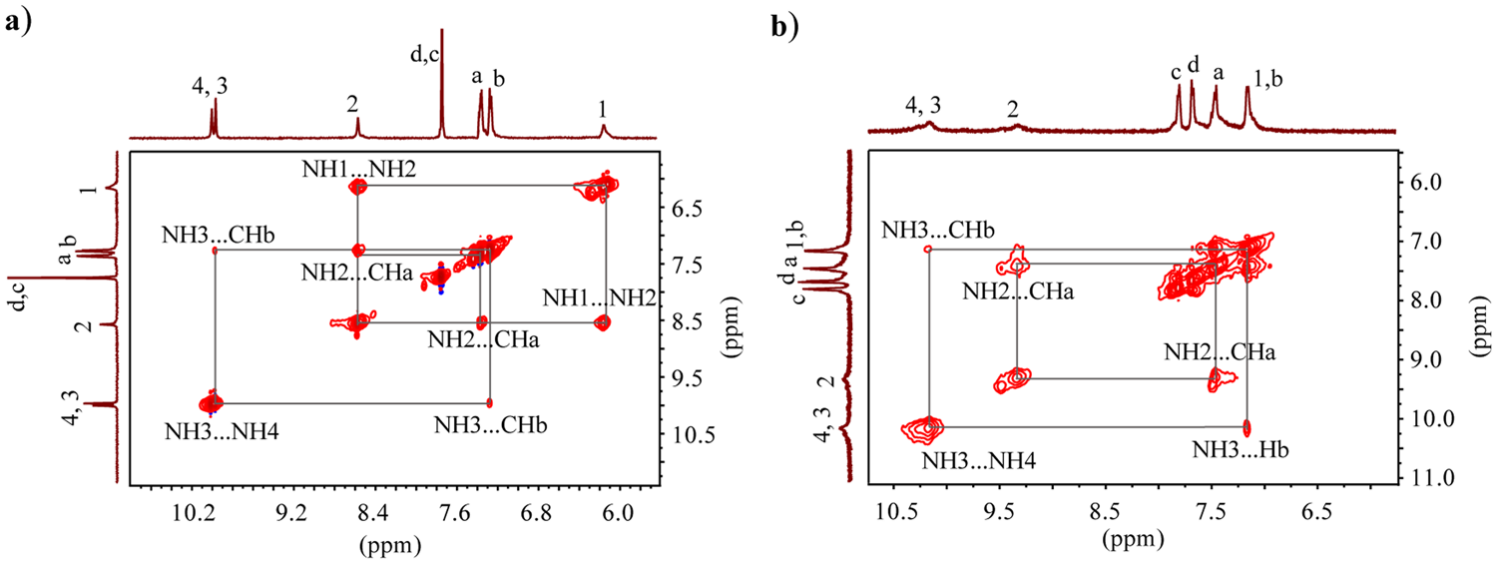
2D NOESY NMR experiment of (**a**) Free **L**, and (**b**) **L** in the presence of one equivalent of sulfate anion in DMSO-*d*_*6*_ at room temperature.

**Figure 5:**
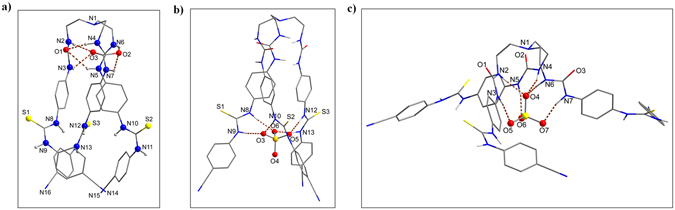
Optimized structures of (**a**) **L**, (**b**) thiourea-bound 1:1 complex [**L**(SO_4_)]^2−^, and (**c**) urea-bound 1:1 complex [**L**(SO_4_)]^2−^, calculated at the M06-2X/6-31G(d,p) level of theory.

The solution binding mode of **L** for sulfate anion was further evaluated by 2D NOESY NMR experiments (Fig. 4), as reported before by us^16^ and others^17, 50^. As shown in Fig. 4a, the free receptor of **L** shows two strong cross peaks for NH1$\cdots$NH2 and NH3$\cdots$NH4 of urea and thiourea moieties, respectively; indicating that these protons are close in space^16^. In addition, two strong couplings for NH2$\cdots$CHa and NH3$\cdots$CHb with aromatic protons were observed. However, the addition of two equivalents of sulfate anions to **L** resulted in the complete loss of NH1$\cdots$NH2 contacts, implying a possible rotation of the two sites of a thiourea unit in order to bind a sulfate anion. On the other hand, the NH3$\cdots$NH4 contacts from urea groups were retained (Fig. 4b), suggesting that these protons remain in a close distance after the encapsulation of sulfate. Indeed, as shown in the optimized structure of the sulfate complexes (Fig. 5b,c, discussed later), the NH sites of a single urea are twisted to bind two oxygen atoms of sulfate inside the *inner cleft*, while this is not the case for thiourea groups, showing the respective sites bonded to a single oxygen atom.

### Computational studies

In an effort to understand the interactions and structural aspects of the new receptor with sulfate, theoretical calculations based on density functional theory (DFT) were performed with hybrid meta-exchange correlation functional M06-2X^51^, using the Gaussian 09 package of programs^52^. Our previous work has shown that the M06-2X functional accurately predicts the binding energy trends for non-covalent interactions between anions and organic receptors^33^. To this end, the initial equilibrium geometry for the free receptor **L** was first optimized at the M06-2X/6-31G(d,p) level of theory^53^. From this equilibrium geometry, the sulfate anion with different orientations was placed in a single (*inner* or *outer*) cleft or both clefts, and molecular geometries of the various sulfate-bound complexes were fully optimized at the M06-2X/6-31G(d,p) level of theory and corrected for zero-point energies (ZPE) in gas phase as well as in a solvent phase to approximate a DMSO environment (dielectric constant = 46.8) using a polarizable continuum model (PCM). With the optimized geometry, the binding energies of **L** for SO_4_^2−^ were calculated using the equation: *ΔE* = *E*(complex) − [*E*(receptor) + *E*(anion)].

As shown in Fig. 5a, the optimized structure of the receptor adopts a perfect *C*_*3*_ symmetric cone shape, due to the presence of three identical arms linked to the tertiary amine. The *inner cleft* of the receptor is decorated with six intra-molecular H-bonds, where each oxygen atom of one urea group is H-bonded with both NH of the adjacent urea unit. Further, all three NH groups of the *outer cleft* are pointed inside the cavity, making it a preferred binding site for a *C*_*3*_ symmetric sulfate anion. With this optimized geometry, we first attempted to organize all NH groups of **L** around a tetrahedral sulfate; however, due to the lack of complementarity, the receptor could not be optimized with a single anion bonded to both clefts simultaneously. Therefore, we proceeded to optimize with one sulfate added separately at each cleft or two sulfates at both clefts of **L**. The optimized structure of the thiourea-bound sulfate complex as shown in Fig. 5b, reveals that the sulfate binds to the *outer cleft* through a total of six NH···O bonds (NH···O = 2.78–2.93 Å). The calculated binding energy of this complex was found to be −203 kcal/mol in gas phase, while it was much lower (−96 kcal/mol) in solvent phase, due to the polarity effect of DMSO solvent included in the calculations^33^.

In contrast to the thiourea-bound sulfate complex, the receptor significantly deformed in the urea-bound sulfate complex (Fig. 5c) to encapsulate a sulfate anion within its *inner cleft*, yielding the binding energies as −151 and −77 kcal/mol in gas and solvent phase, respectively. The calculated binding energies for thiourea-bound complex (*ΔE* = −203 kcal/mol) and for urea-bound complex (*ΔE* = −151 kcal/mol) are comparable to our previous report on sulfate binding with a *tris*-thiourea (*ΔE* = −200 kcal/mol) and a *tris*-urea (*ΔE* = −173 kcal/mol) in gas phase^33^. The higher binding energy for the thiourea-bound complex (Fig. 5b) than that for the urea-bound complex (Fig. 5c) demonstrates that the *outer cleft* is the preferential binding site for the first sulfate, which is in agreement with the experimental results. As mentioned previously, these results further support our assumption that the binding of the first sulfate at the *outer cleft* (Fig. 5b) may allow the second sulfate to bind at the *inner cleft*.

**Figure 6:**
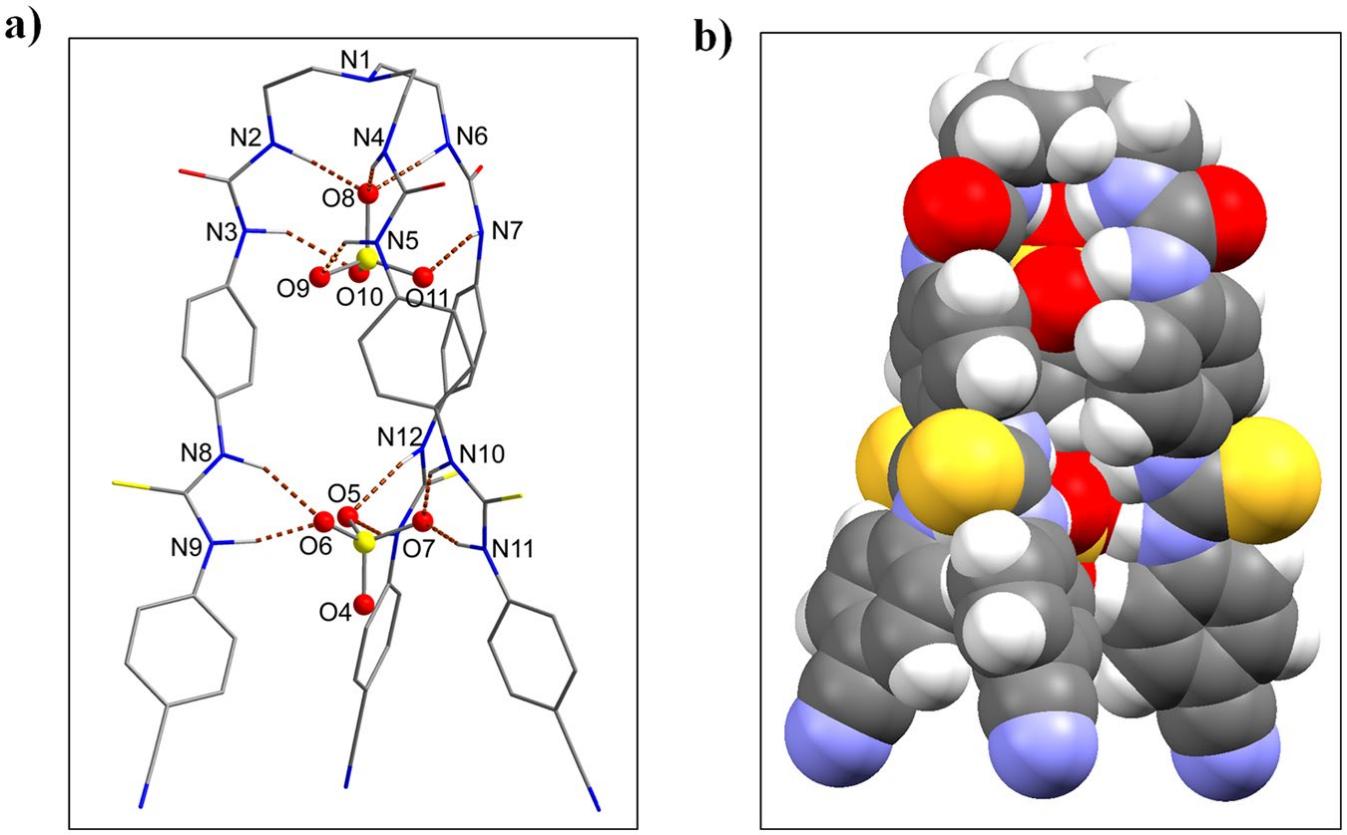
Optimized structures of 1:2 complex [**L**(SO_4_)_2_]^4−^ complex showing (**a**) perspective view, and (**b**) space filling model, calculated at the M06-2X/6-31G(d,p) level of theory.

Considering that the first binding occurs at the *outer cleft* (thiourea groups), followed by the second binding at the *inner cleft* (urea groups), as proposed by NMR titration studies, we proceeded to re-optimize the receptor with two sulfate ions by incorporating both clefts, each with a single sulfate. The calculated binding energies were found to be −161 and −87 kcal/mol in gas and solvent phase, respectively. The optimized structure, as displayed in Fig. 6, reveals that both the *inner cleft* and the *outer cleft* are occupied by sulfate anions that are bound through strong H-bonding interactions (NH···O < 2.94 Å), thereby overcoming the expected electrostatic repulsion due to the encapsulation of two anions in a single molecule. It is noteworthy that the receptor, in a 1:1 complex (urea- or thiourea-bound sulfate), readjusted its geometry to implement maximum interactions for sulfate that is bonded through six NH···O bonds (see bond distances in Table 2). While the thiourea-bound complex adopted a perfect *C*_*3*_ symmetry, leaving the urea-cleft open for a second sulfate (see Fig. 5b); the urea-bound complex deviated from its *C*_*3*_ conformation, adopting a folded umbrella that could not allow to bind another sulfate due to the nonexistence of the outer cavity (Fig. 5c). On the other hand, the receptor is stabilized with two sulfates, each with six NH···O bonds from six NH binding sites from a single cleft (*inner* or *outer*), creating a perfect *C*_*3*_ symmetric 1:2 complex.

**Table 2:** Hydrogen parameters (Å, °) for the sulfate complexes of **L** calculated with DFT at M06-2X/6-31G(d,p).

Complex	NH $\cdots$ O	NH $\cdots$ O	H$\cdots$O	∠DHO
Thiourea-bound [**L**(SO_4_)]^2‒^	N8H $\cdots$ O6	2.935	1.908	174.1
	N9H $\cdots$ O3	2.784	1.758	170.4
	N10H $\cdots$ O3	2.935	1.908	174.1
	N11H $\cdots$ O5	2.784	1.758	170.4
	N12H $\cdots$ O5	2.935	1.908	174.1
	N13H $\cdots$ O6	2.784	1.758	170.4
Urea-bound [**L**(SO_4_)]^2‒^	N2H $\cdots$ O4	2.789	1.799	160.8
	N3H $\cdots$ O5	2.847	1.839	164.6
	N4H $\cdots$ O4	2.778	1.786	161.1
	N5H $\cdots$ O6	2.869	1.869	162.7
	N6H $\cdots$ O4	2.775	1.785	160.6
	N7H $\cdots$ O7	2.869	1.866	163.7
[**L**(SO_4_)_2_]^4‒^	N2H $\cdots$ O8	2.678	1.643	168.8
	N3H $\cdots$ O10	2.883	1.989	144.4
	N8H $\cdots$ O6	2.934	1.998	150.3
	N9H $\cdots$ O6	2.701	1.675	166.2
	N4H $\cdots$ O8	2.678	1.643	168.8
	N5H $\cdots$ O9	2.883	1.989	144.4
	N10H $\cdots$ O7	2.934	1.998	150.3
	N11H $\cdots$ O7	2.701	1.675	166.2
	N6H $\cdots$ O8	2.678	1.643	168.8
	N7H $\cdots$ O11	2.883	1.989	144.4
	N12H $\cdots$ O5	2.934	1.998	150.3
	N13H $\cdots$ O5	2.701	1.675	166.2

## Conclusion

We have designed and synthesized a novel *para*-phenylene-bridged hexafunctional tripodal receptor consisting of two different functionalized clefts (urea-based *inner cleft* and thiourea-based *outer cleft*). As demonstrated by experimental studies and theoretical calculations, the receptor can effectively bind sulfate anions in a two-step binding process, leading to a well-defined 1:2 stoichiometric complex that is stabilized through complementary H-bonding interactions. Our results suggest that the unique combination of two different functionalities makes the receptor ideal to bind the first sulfate at the thiourea-based *outer cleft* and the second sulfate at the urea-based *inner cleft*. The preferred binding at the *outer cleft* is due to the enhanced H-bonding ability as well as of the structural complementarity of thiourea functionalities, leading to stronger interactions with the anion than those with its urea analogue. This binding propagation was further supported by DFT calculations, illustrating that the thiourea-bound complex is energetically more favorable than the urea-bound complex. Therefore, we conclude that the binding of one sulfate at the *outer cleft* assists the receptor to bind the second sulfate at the *inner cleft*. To the best of our knowledge, such an assembled multifunctional anion receptor with the unique combination of a *urea*-*based cleft* and a *thiourea*-*based cleft* has not been reported previously. Understanding and being able to accurately predict the interactions between synthetic receptors and guests is a key step towards elucidating the complex mechanisms in living systems. Taken together, the results from our study may be useful in developing highly organized molecular receptors for extraction, catalysis and drug design for environmental and biomedical applications.

## Methods

### General

All reagents and solvents were purchased as reagent grade and were used without further purification. Nuclear magnetic resonance (NMR) spectra were recorded on a Varian Unity INOVA 500 FT-NMR. Chemical shifts for samples were measured in DMSO-*d*_*6*_ and calibrated against sodium salt of 3-(trimethylsilyl) propionic-2,2,3,3-*d*_*4*_ acid (TSP) as an external reference in a sealed capillary tube. NMR data were processed and analyzed with MestReNova Version 6.1.1-6384. The melting point was determined on a Mel-Temp (Electrothermal 120 VAC 50/60 Hz) melting point apparatus and was uncorrected. Elemental analysis was carried out by ECS 4010 Analytical Platform (Costech Instrument) elemental analyzer at Jackson State University.

### *Tris*-(4-nitrophenyl)-urea (2)

*Tris*(2-aminoethyl)amine **1** (1.04 mL, 6.95 mmol) was mixed with three equivalents of *p*-nitrophenyl isocyanate (3.47 g, 21.14 mmol) in CH_2_Cl_2_ under reflux for 24 hours. A yellow precipitate was formed when the reaction mixture was cooled down. The precipitate was collected by filtration and washed with dichloromethane and diethyl ether solvent. The compound was dried over vacuum to give the analytically pure **2** as a yellow solid. Yield: 3.8 g (86%); ^1^H NMR (500 MHz, DMSO-*d*_*6*_): δ 9.35 (s, 3H, Ar-N*H*), 8.07 (d, J = 8.6 Hz, 6H, Ar*H*), 7.57 (d, J = 8.5 Hz, 6H, Ar*H*), 6.43 (s, 3H, CH_2_N*H*), 3.22 (br, 6H, NHC*H*_2_), 2.62 (br, 6H, NC*H*_2_); ^13^C NMR (125 MHz, DMSO-*d*_*6*_): δ 179.7, 145.9, 141.5, 124.35, 120.2, 51.2, 41.47.

### *Tris*-(4-aminophenyl)-urea (3)

To a suspension of **2** in ethanol (1.0 L) containing 10% Pd/C (1.0 g) as a catalyst, hydrazine monohydrate (12.0 mL) was added drop-wise at room temperature. After refluxing for 4 hrs, the reaction mixture was filtrated through celite to remove Pd/C. The filtrate thus collected was evaporated to dryness. The white solid was washed with diethyl ether several times and dried over vacuum to **3**. Yield: 2.9 g (89%); ^1^H NMR (500 MHz, DMSO-*d*_*6*_): δ 8.03 (s, 3H, Ar-N*H*), 6.97 (d, J = 7.9 Hz, 6H, Ar*H*), 6.44 (d, J =  8.0 Hz, 6H, Ar*H*), 6.03 (s, 3H, CH_2_N*H*), 4.66 (s, 6H, Ar-N*H*), 3.11 (br, 6H, NHC*H*_2_), 2.50 (br, 6H, NC*H*_2_); ^13^C NMR (125 MHz, DMSO-*d*_*6*_): δ 155.9, 143.4, 129.5, 120.4, 114.1, 54.1, 37.5.

### Tripodal 4-amino phenyl *tris*-(4-cyanophenyl)-hexafunctional urea/thiourea ligand (L)

The compound **3** (2.5 g, 4.55 mmol) was added to three equivalent *p*-cyanophenyl isothiocyanate (2.2 g, 13.73 mmol) in methanol and the mixture was refluxed for overnight at 100–130 °C. A white precipitate was formed when the reaction mixture was cooled down. The precipitate was collected by filtration and washed with methanol and diethyl ether. The compound was dried over vacuum to give the hexafunctional receptor **L** as a white solid. Yield: 2.5 g (53%); mp: 245 °C; ^1^H NMR (500 MHz, DMSO-*d*_*6*_): δ 10.02 (s, 3H, ArN*H*CS), 9.99 (s, 3H, ArN*H*CS), 8.58 (s, 3H, CON*H*Ar), 7.75 (s, 12H, Ar*H*), 7.36 (d, J = 8.7 Hz, 6H, Ar*H*), 7.26 (d, 8.6 Hz, 6H, Ar*H*), 6.16 (s, 3H, CH_2_N*H*CO), 3.19 (d, J = 4.8 Hz, 6H, NHC*H*_2_), 2.60 (s, 6H, NC*H*_2_); ^13^C NMR (125 MHz, DMSO-*d*_*6*_): δ 179.4 (*C*=S), 155.3 (*C*=O), 144.3 (Ar*C*), 137.9 (Ar*C*), 132.7 (Ar*C*), 132.1 (Ar*C*), 124.9 (Ar*C*), 122.4 (Ar*C*), 119.2 (Ar*C*), 117.9 (Ar*C*N), 105.2 (Ar*C*), 54.0 (NH*C*H_2_); ESI-MS (ESI^+^, CH_3_OH), m/z calcd for [M + H]^+^ 1029.32, found 1029.14; analysis (calcd., found for C_51_H_48_N_16_O_3_S_3_): C, 59.52, 59.29), H (4.70, H, 4.64), N (21.77, 21.69).

### ^1^H NMR Binding Studies

Binding constants were obtained by ^1^H NMR (Varian Unity INOVA 500 FT-NMR) titrations of **L** with the oxoanions (NO_3_^—^, ClO_4_^—^, H_2_PO_4_^—^, HSO_4_^—^, SO_4_^2—^) at neutral pH. Initial concentrations were [ligand]_0_ = 2 mM, and [anion]_0_ = 20 mM. Each titration was performed by 13 measurements at room temperature. The association constant *K* was calculated by fitting of several independent NMR signals using a 1:2 (**L**:anion) binding model using the EQNMR program. Error limit in *K* was less than 15%.

### Computational studies

Interaction energies and geometry optimization of sulfate complexes were performed with density functional theory (DFT) calculations^51^. All calculations were carried out using Gaussian 09 package of programs^52^.

### Data Availability

All data generated or analysed during this study are included in this published article and its Supplementary Information files.

## Electronic supplementary material

Supplementary Information
