# Supplementary material for: Remarkable hexafunctional anion receptor with operational urea-based inner cleft and thiourea-based outer cleft: Novel design with high-efficiency for sulfate binding
Source: Sci Rep. 2017 Jul 20;7:6032. doi: 10.1038/s41598-017-05831-x (PMC5519707; doi:10.1038/s41598-017-05831-x)
Supplement: Supplementary file 1 — Supplementary Information [file 41598_2017_5831_MOESM1_ESM.doc]

**Supplementary Information**

Remarkable hexafunctional anion receptor with operational urea-based *inner cleft* and thiourea-based *outer cleft*: Novel design with high-efficiency for sulfate binding

Maryam Emami Khansari, Ali Mirchi, Avijit Pramanik, Corey R. Johnson, Jerzy Leszczynski*and Md. Alamgir Hossain*

Department of Chemistry and Biochemistry, Jackson State University, Jackson, MS 39217, USA

*Correspondence and requests for materials should be addressed to

M.A.H. (alamgir.hossain@jsums.edu) or J.L. (jerzy@icnanotox.org)

**
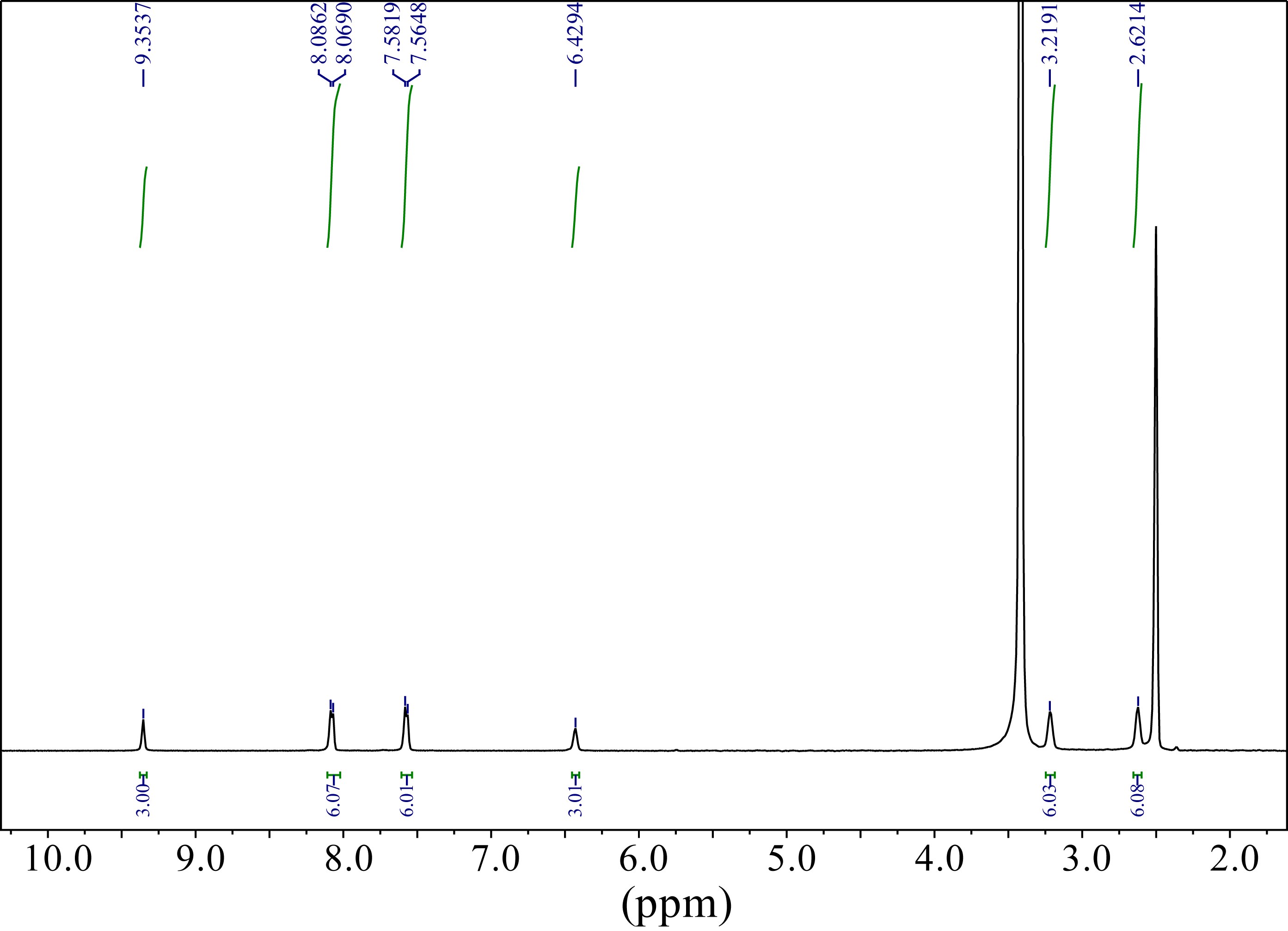
**

**Figure S1.** ^1^H NMR spectrum of **2** in DMSO-*d_6_*.


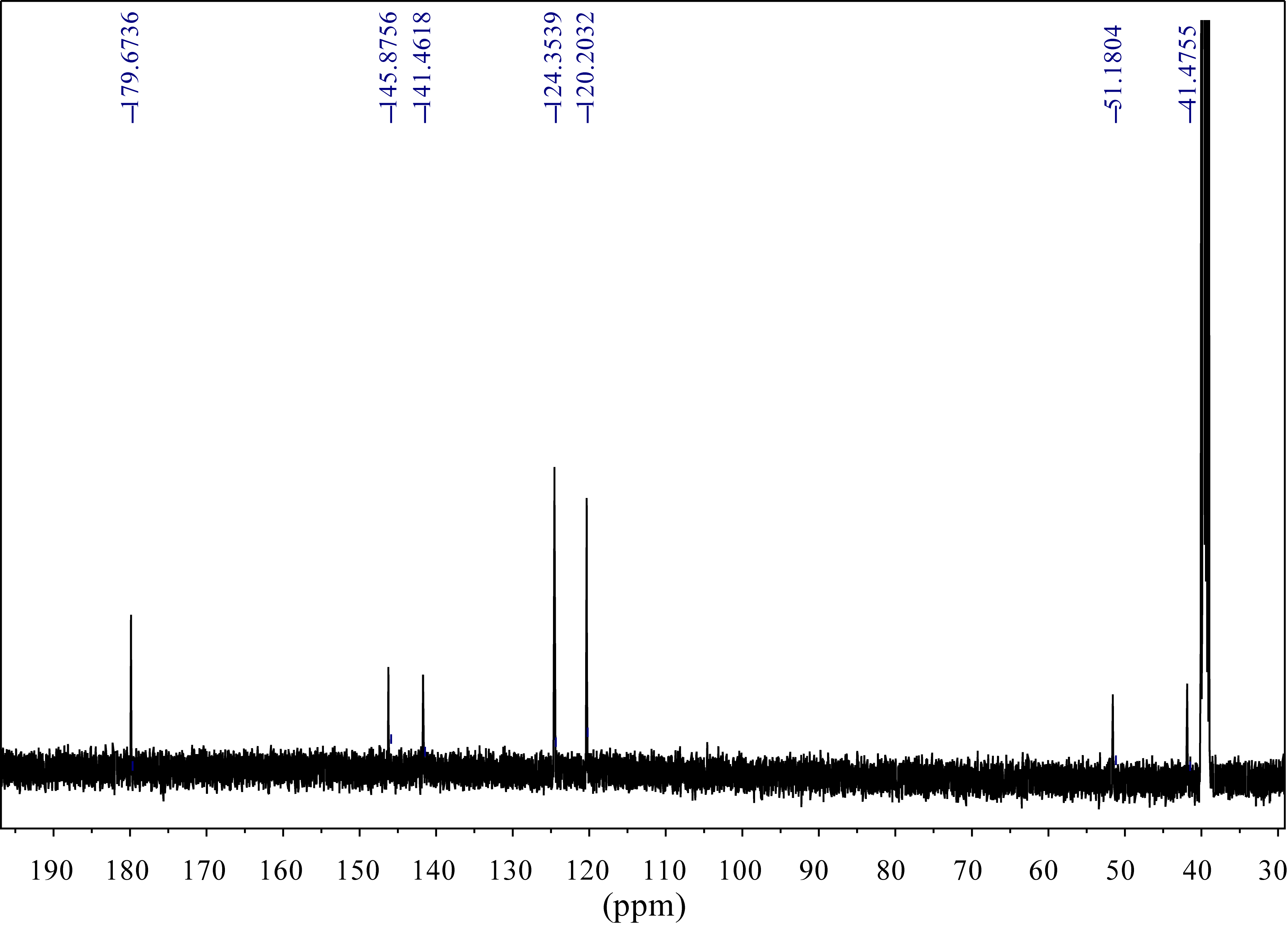


**Figure S2.** ^13^C NMR spectrum of **2** in DMSO-*d_6_*.


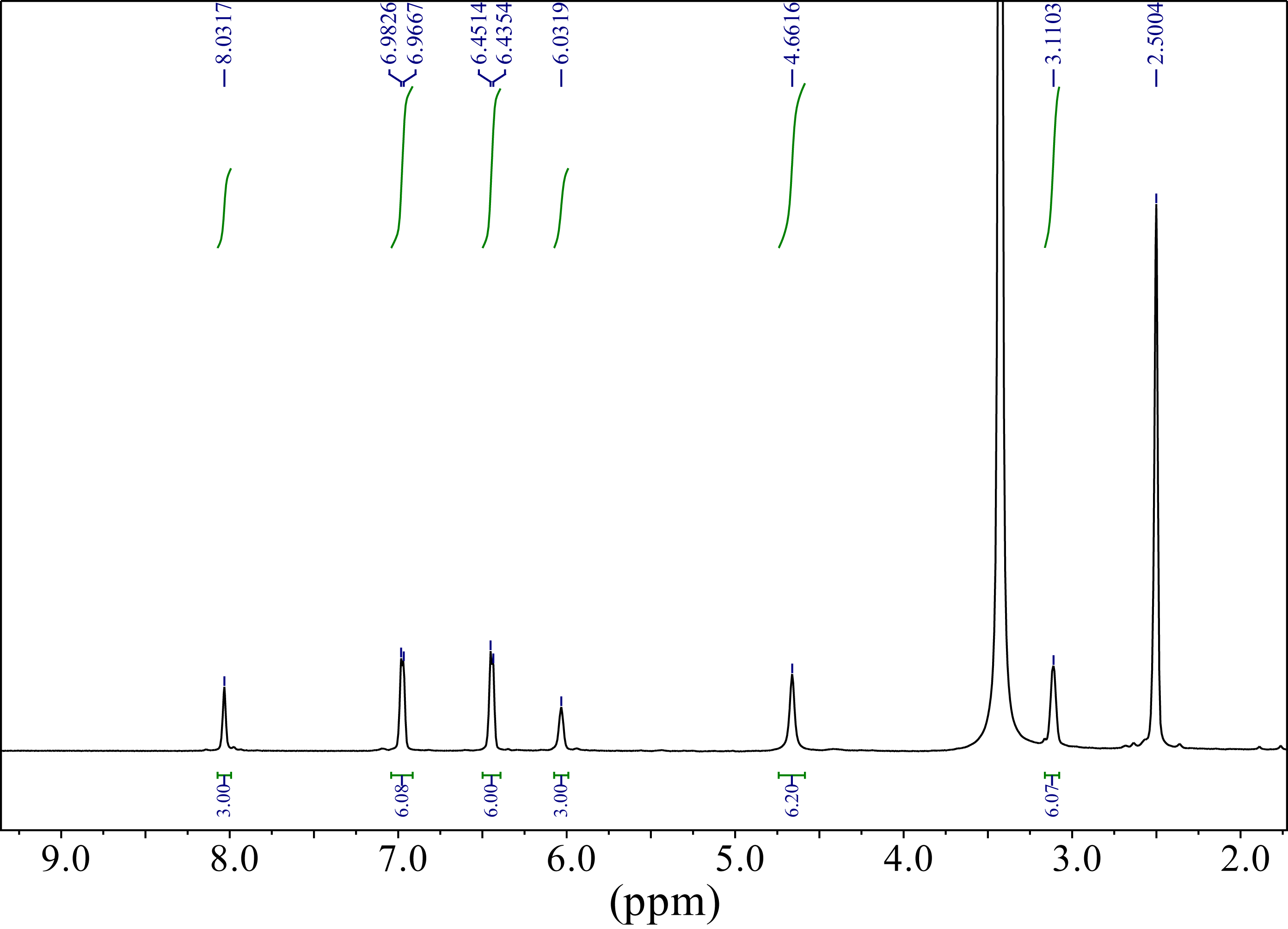


**Figure S3.** ^1^H NMR spectrum of **3** in DMSO-*d_6_*.


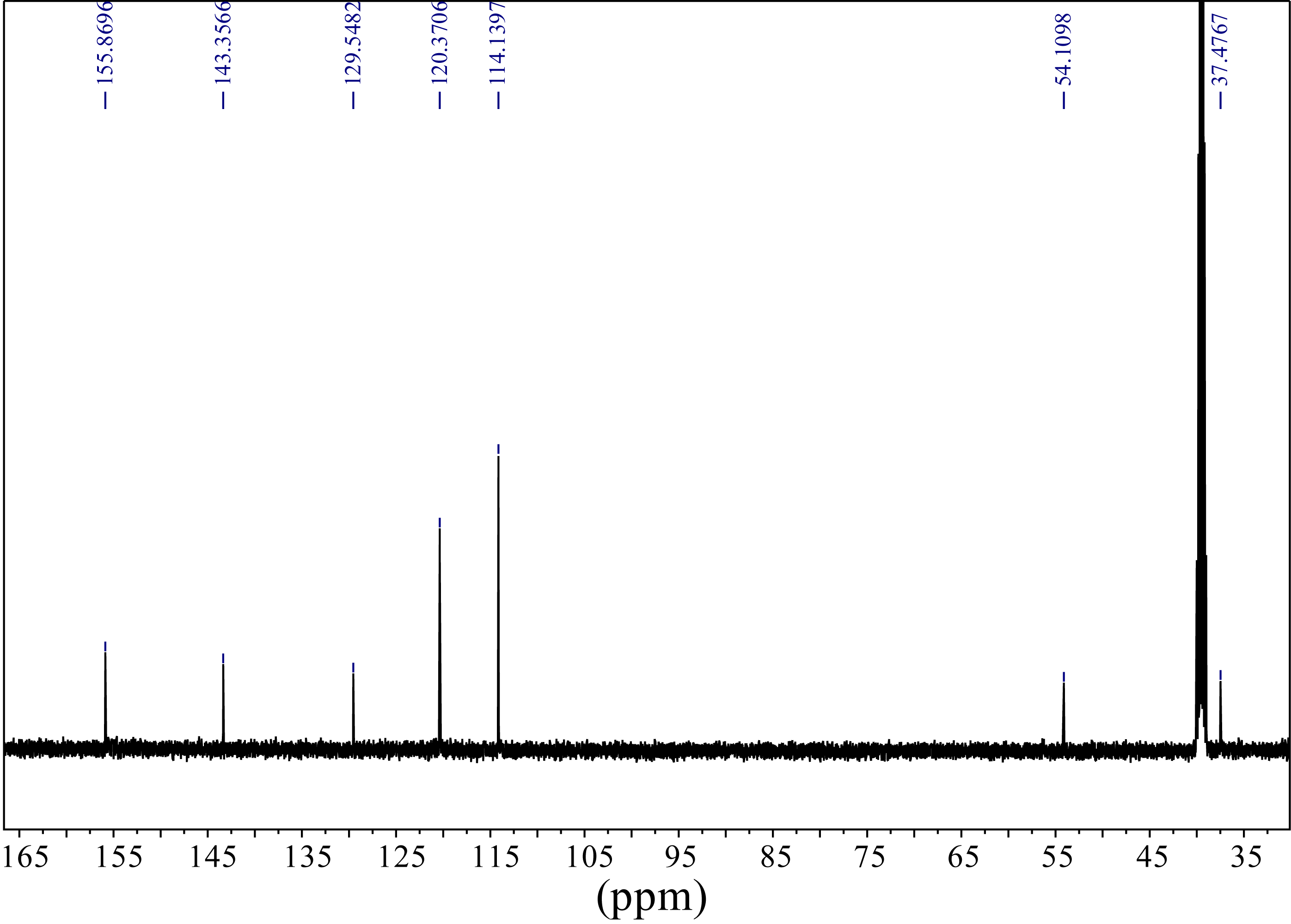


**Figure S4.** ^13^C NMR spectrum of **3** in DMSO-*d_6_*.


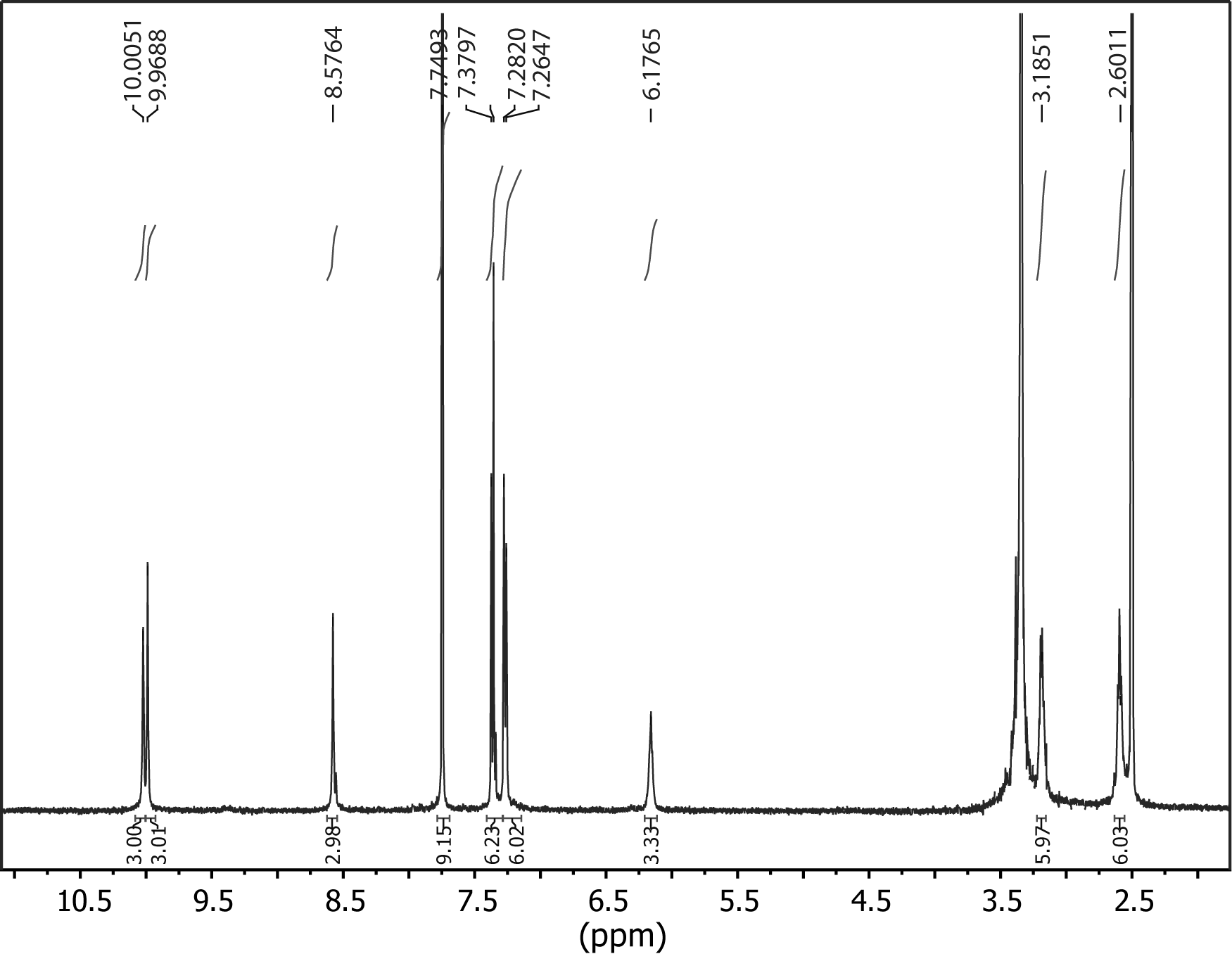


**Figure S5.** ^1^H NMR spectrum of **L** in DMSO-*d_6_*.


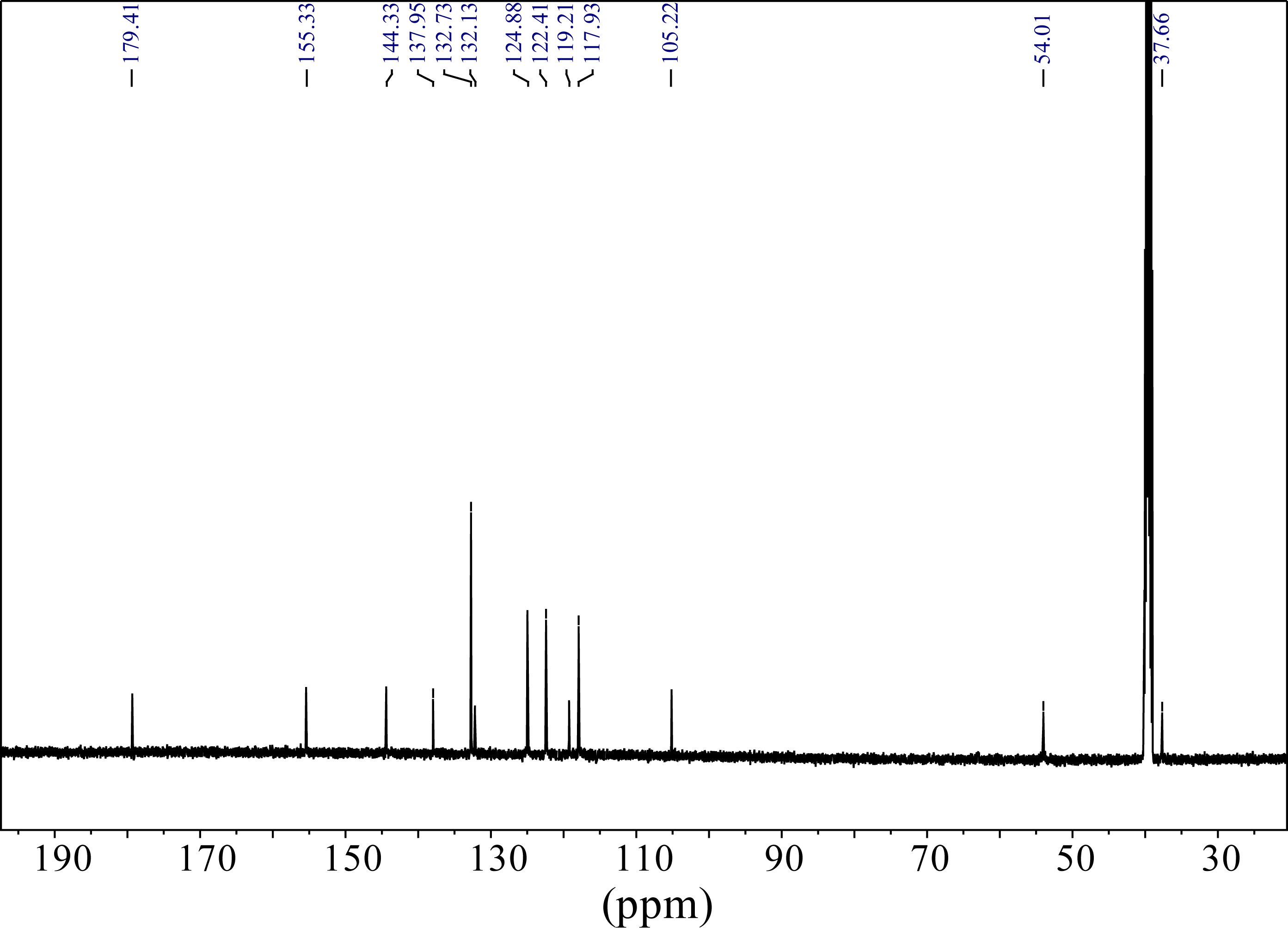


**Figure S6.** ^13^C NMR spectrum of **L** in DMSO-*d_6_*.


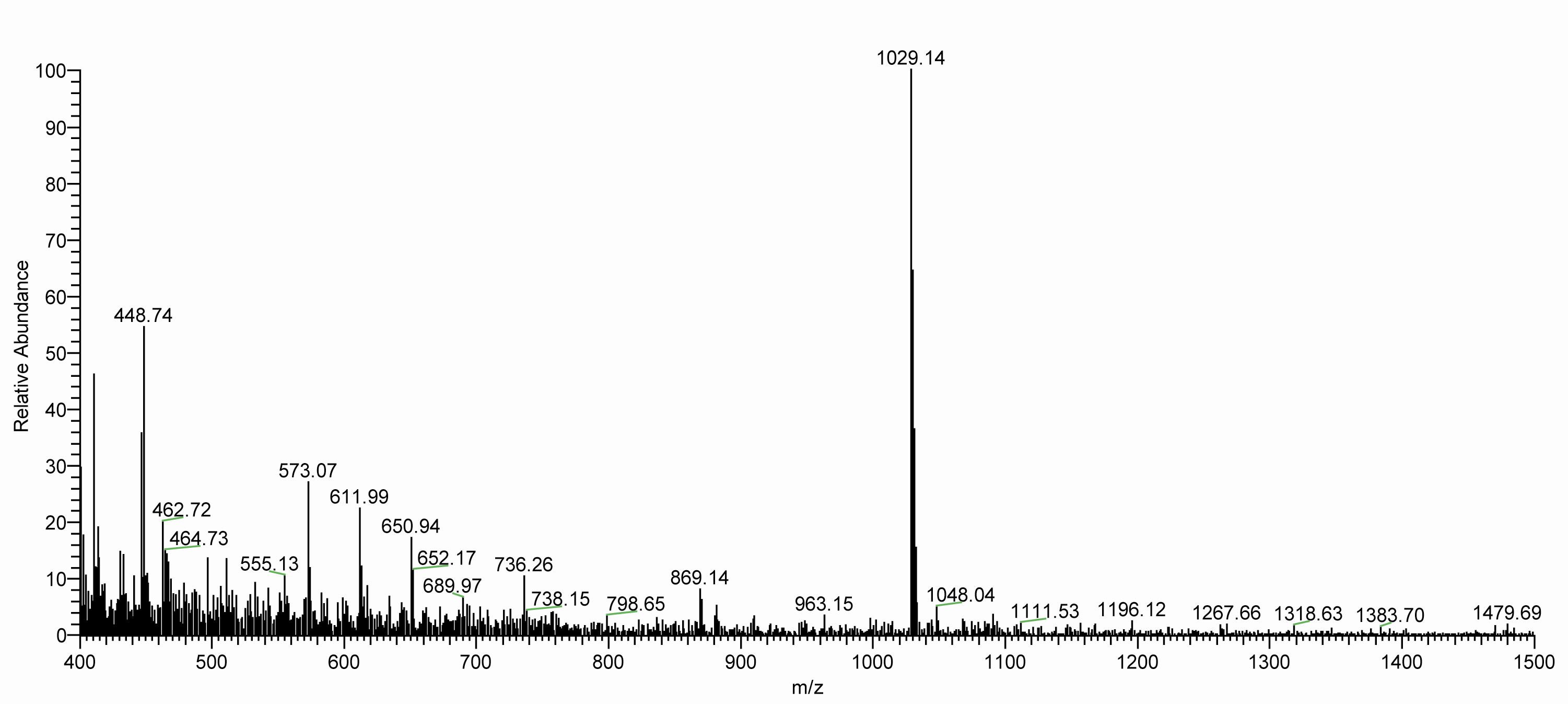


**Figure S7.** ESI-MS spectrum of **L**.

|  |
| --- |

**Figure S8.** Titration curves of **L** (2 mM) with SO_4_^2-^ (20 mM) showing changes in the chemical shifts of NH2, analyzed with a 1:2 (**L**:anion) binding model using the EQNMR program (observed values shown by the green circles and calculated values represented by the line).


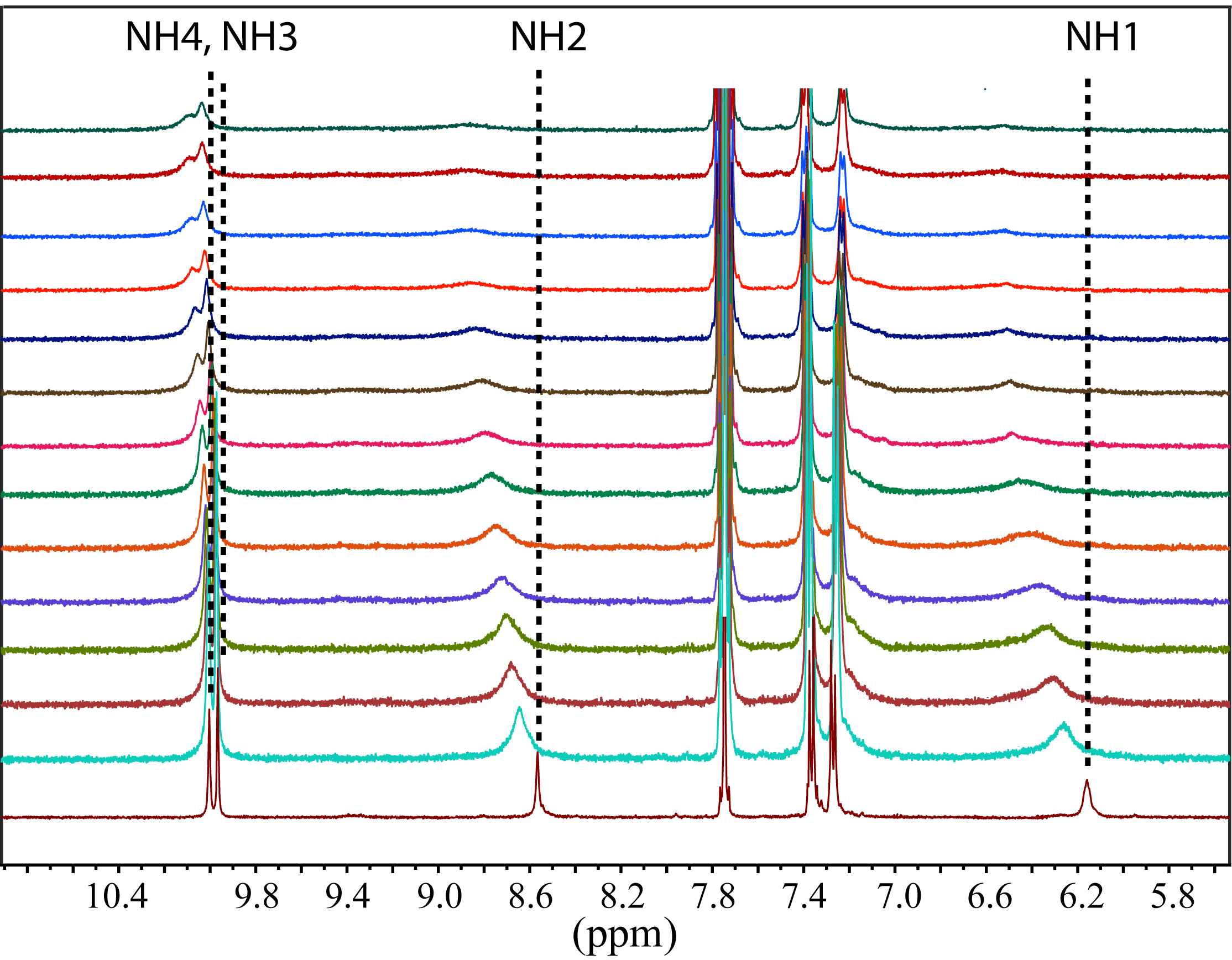


**Figure S9.** (a) Partial ^1^H NMR spectra of **L** (2mM) with an increasing amount of HSO_4_^-^ (R = 0 -10) in DMSO-*d_6_* (H1=ArNHCS, H2=CSNHAr, H3= ArNHCO, H4= CONHCH_2_).

|  |
| --- |

**Figure S10.** Titration curves of **L** (2 mM) with HSO_4_^-^ (20 mM) showing changes in the chemical shifts of NH2, analyzed with a 1:2 (**L**:anion) binding model using the EQNMR program (observed values shown by the green circles and calculated values represented by the line).

**Figure S11.** Partial ^1^H NMR spectra of **L** (2mM) with an increasing amount of H_2_PO_4_^-^ (R = 0 -10) in DMSO-*d_6_* (H1=ArNHCS, H2=CSNHAr, H3= ArNHCO, H4= CONHCH_2_).

**Figure S12.** Titration curves of **L** (2 mM) with H_2_PO_4_^-^ (20 mM) showing changes in the chemical shifts of NH2, analyzed with a 1:2 (**L**:sulfate) binding model using the EQNMR program (observed values shown by the green circles and calculated values represented by the line).


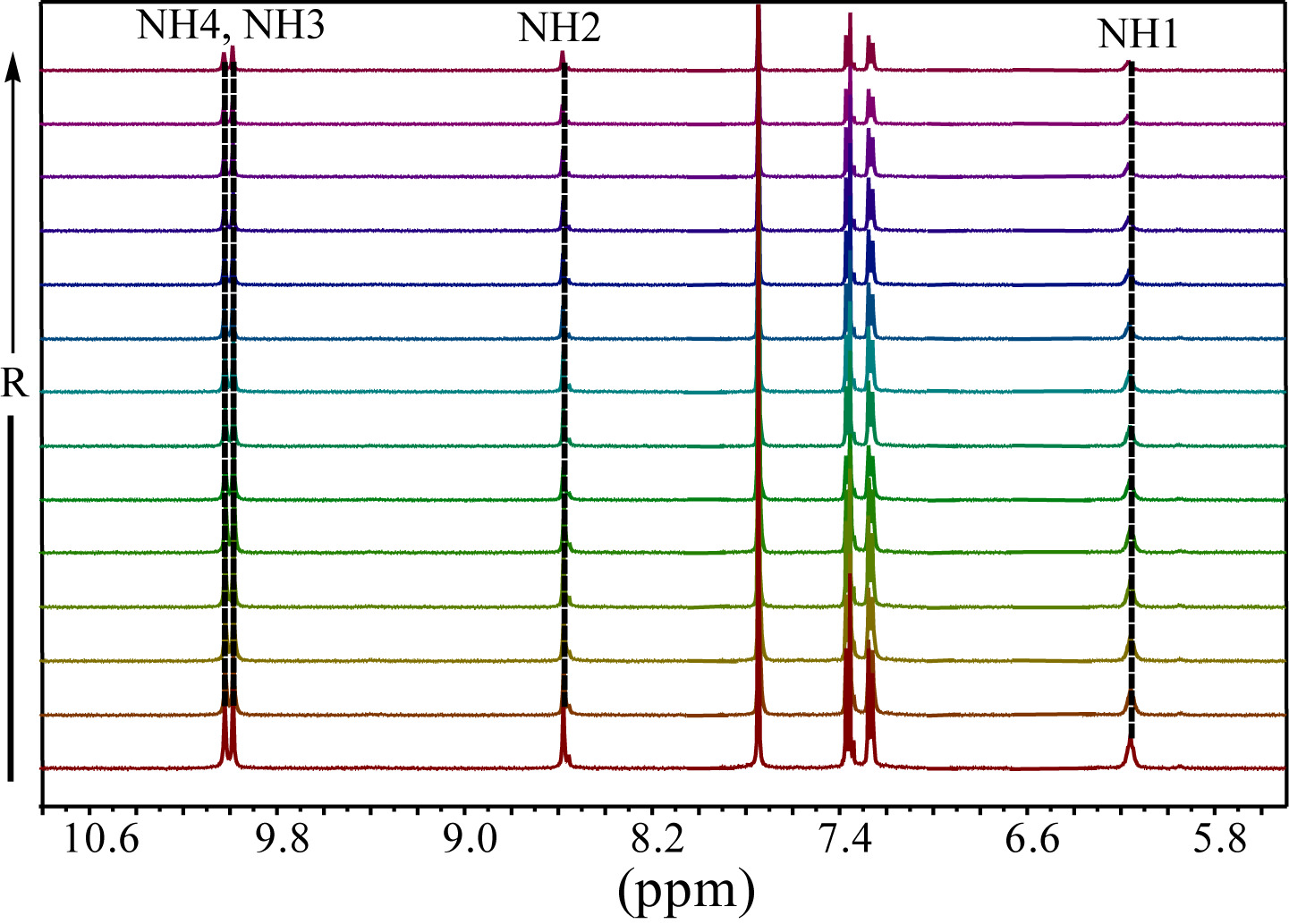


**Figure S13.** Partial ^1^H NMR spectra of **L** (2mM) with an increasing amount of NO_3_^-^ (R = 0 -10) in DMSO-*d_6_* (H1=ArNHCS, H2=CSNHAr, H3= ArNHCO, H4= CONHCH_2_).


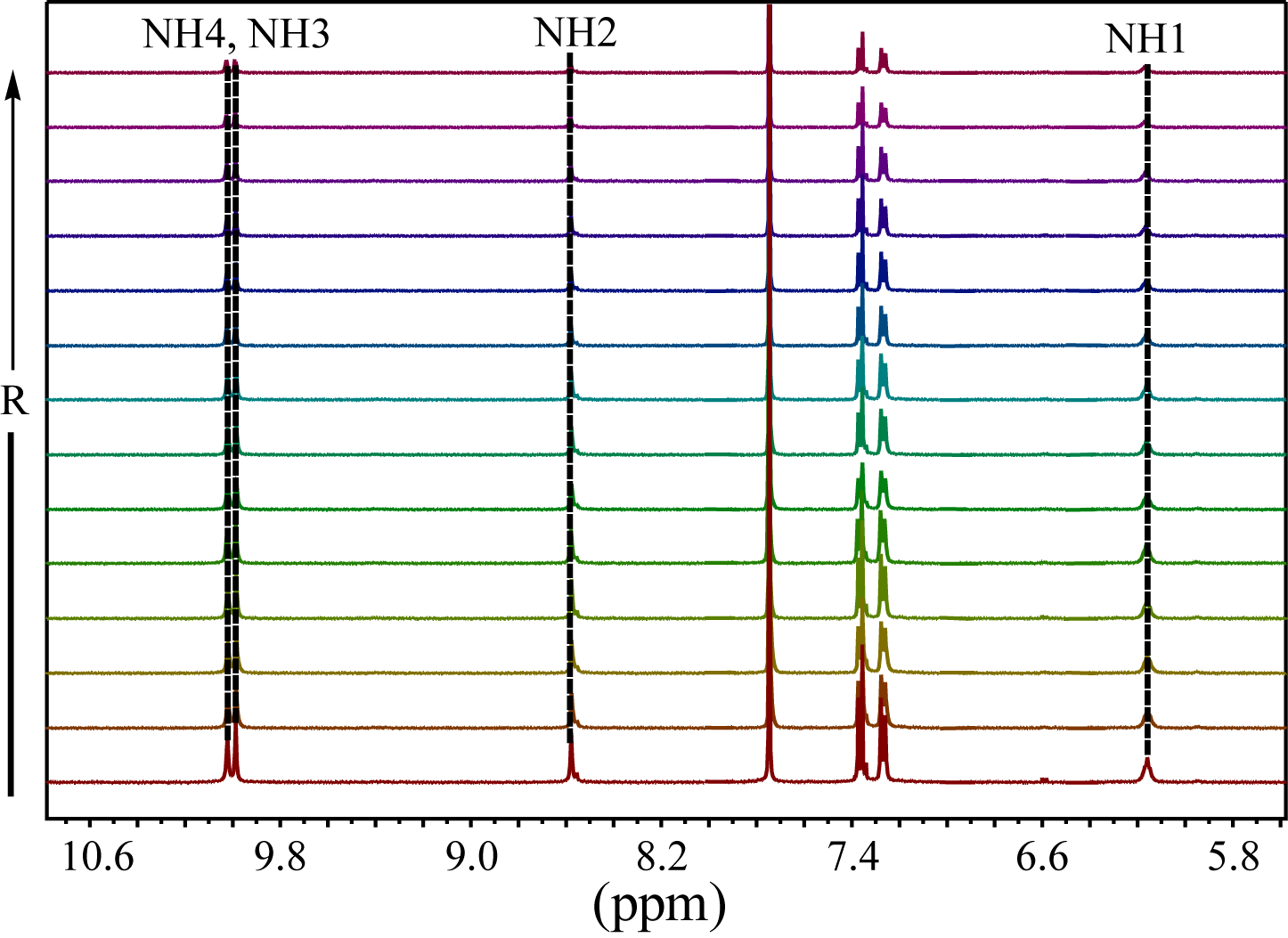


**Figure S14.** Partial ^1^H NMR spectra of **L** (2mM) with an increasing amount of ClO_4_^-^ (R = 0 -10) in DMSO-*d_6_* (H1=ArNHCS, H2=CSNHAr, H3= ArNHCO, H4= CONHCH_2_).

**Computational Data**

**Table S1.** Cartesian coordinates (in Å) of **L** calculated at the M06-2X/6-31G(d,p) level of theory in gas phase.

Total energy: -4267.44062670 au.

---------------------------------------------------------------------

Center Atomic Coordinates (Angstroms)

No. Number X Y Z

-----------------------------------------------------------

1 7 0.000000 0.000000 7.581073

2 6 0.259323 1.368912 7.984900

3 1 0.340237 1.478411 9.083935

4 1 -0.578157 1.988825 7.658951

5 6 1.530265 1.941849 7.342232

6 1 1.472647 3.033073 7.385434

7 1 2.423239 1.644408 7.896514

8 7 1.742269 1.544017 5.964820

9 1 2.279848 0.699757 5.788173

10 6 0.888539 1.959366 4.994534

11 8 -0.078952 2.696427 5.214287

12 7 1.225226 1.526143 3.727280

13 1 1.950974 0.820451 3.661154

14 6 0.791246 2.077479 2.513725

15 6 -0.053452 3.193514 2.409013

16 1 -0.403279 3.689592 3.302802

17 6 -0.449564 3.661248 1.158214

18 1 -1.112316 4.514453 1.091320

19 6 0.000000 3.041075 -0.007023

20 6 0.871553 1.956015 0.098780

21 1 1.246844 1.471139 -0.800589

22 6 1.264475 1.482726 1.337742

23 6 -1.315174 -0.459876 7.984900

24 1 -1.450460 -0.444552 9.083935

25 1 -1.433295 -1.495111 7.658951

26 6 -2.446823 0.354324 7.342232

27 1 -3.363042 -0.241187 7.385434

28 1 -2.635719 1.276382 7.896514

29 7 -2.208293 0.736841 5.964820

30 1 -1.745931 1.624527 5.788173

31 6 -2.141130 -0.210185 4.994534

32 8 -2.295698 -1.416588 5.214287

33 7 -1.934292 0.298005 3.727280

34 1 -1.686018 1.279368 3.661154

35 6 -2.194773 -0.353501 2.513725

36 6 -2.738939 -1.643048 2.409013

37 1 -2.993641 -2.194045 3.302802

38 6 -2.945952 -2.219957 1.158214

39 1 -3.353473 -3.220520 1.091320

40 6 -2.633648 -1.520538 -0.007023

41 6 -2.129735 -0.223221 0.098780

42 1 -1.897466 0.344229 -0.800589

43 6 -1.916316 0.353705 1.337742

44 6 1.055851 -0.909036 7.984900

45 1 1.110223 -1.033860 9.083935

46 1 2.011452 -0.493714 7.658951

47 6 0.916558 -2.296173 7.342232

48 1 1.890395 -2.791886 7.385434

49 1 0.212480 -2.920790 7.896514

50 7 0.466024 -2.280858 5.964820

51 1 -0.533916 -2.324284 5.788173

52 6 1.252591 -1.749180 4.994534

53 8 2.374650 -1.279839 5.214287

54 7 0.709066 -1.824149 3.727280

55 1 -0.264956 -2.099819 3.661154

56 6 1.403527 -1.723978 2.513725

57 6 2.792391 -1.550466 2.409013

58 1 3.396919 -1.495546 3.302802

59 6 3.395515 -1.441290 1.158214

60 1 4.465789 -1.293933 1.091320

61 6 2.633648 -1.520538 -0.007023

62 6 1.258182 -1.732794 0.098780

63 1 0.650622 -1.815368 -0.800589

64 6 0.651840 -1.836431 1.337742

65 1 -0.415853 -2.008762 1.395719

66 1 1.947566 0.644242 1.395719

67 1 -1.531713 1.364521 1.395719

68 7 3.170388 -1.318098 -1.301846

69 1 2.517416 -0.923970 -1.967690

70 7 -0.443688 3.404686 -1.301846

71 1 -0.458526 2.642132 -1.967690

72 7 -2.726701 -2.086588 -1.301846

73 1 -2.058890 -1.718161 -1.967690

74 6 4.340441 -1.778036 -1.817168

75 6 -0.630396 4.647950 -1.817168

76 6 -3.710044 -2.869914 -1.817168

77 16 5.427409 -2.781803 -1.055617

78 16 -5.122816 -3.309372 -1.055617

79 16 -0.304593 6.091175 -1.055617

80 7 -1.091906 4.650249 -3.114814

81 1 -1.021846 5.558002 -3.554424

82 7 4.573186 -1.379506 -3.114814

83 1 5.324294 -1.894056 -3.554424

84 7 -3.481281 -3.270742 -3.114814

85 1 -4.302448 -3.663946 -3.554424

86 6 -2.319911 -3.147022 -3.896965

87 6 -2.459364 -2.804152 -5.245950

88 6 -1.043032 -3.408211 -3.373569

89 6 -1.345186 -2.712214 -6.064840

90 1 -3.440565 -2.579207 -5.650549

91 6 0.073868 -3.304028 -4.189485

92 1 -0.936464 -3.725490 -2.341436

93 6 -0.072534 -2.957360 -5.538444

94 1 1.060631 -3.517800 -3.791177

95 6 3.885356 -0.435591 -3.896965

96 6 3.658149 -0.727796 -5.245950

97 6 3.473113 0.800813 -3.373569

98 6 3.021439 0.191142 -6.064840

99 1 3.953941 -1.690014 -5.650549

100 6 2.824438 1.715986 -4.189485

101 1 3.694601 1.051743 -2.341436

102 6 2.597416 1.415864 -5.538444

103 1 2.832698 -0.056001 -7.103815

104 1 2.516188 2.677433 -3.791177

105 6 -1.565445 3.582612 -3.896965

106 6 -2.430081 2.607397 -3.373569

107 6 -1.198785 3.531948 -5.245950

108 6 -2.898306 1.588042 -4.189485

109 1 -2.758137 2.673747 -2.341436

110 6 -1.676253 2.521072 -6.064840

111 1 -0.513376 4.269220 -5.650549

112 6 -2.524882 1.541496 -5.538444

113 1 -3.576819 0.840367 -3.791177

114 1 -1.367850 2.481189 -7.103815

115 6 1.927687 2.365353 -6.384095

116 7 1.378562 3.127581 -7.060801

117 6 -3.012299 0.486750 -6.384095

118 7 -3.397845 -0.369921 -7.060801

119 6 1.084612 -2.852102 -6.384095

120 7 2.019283 -2.757660 -7.060801

121 1 -1.464847 -2.425187 -7.103815

---------------------------------------------------------------------

**Table S2.** Cartesian coordinates (in Å) of the thiourea-bound 1:1 complex [**L**(SO_4_)]^2‒^ calculated at the M06-2X/6-31G(d,p) level of theory in gas phase.

Total energy: -4966.55629963 au.

---------------------------------------------------------------------

Center Atomic Coordinates (Angstroms)

No. Number X Y Z

------------------------------------------------------------

1 7 0.000000 0.000000 -8.170471

2 6 1.042659 0.926390 -8.559025

3 1 1.111228 1.049380 -9.660040

4 1 2.000791 0.531486 -8.214814

5 6 0.868370 2.310157 -7.917600

6 1 1.830109 2.828957 -7.971005

7 1 0.145881 2.913018 -8.474444

8 7 0.432013 2.293154 -6.540688

9 1 -0.567959 2.242024 -6.358360

10 6 1.270841 1.812742 -5.567431

11 8 2.407398 1.389912 -5.817104

12 7 0.750310 1.901564 -4.309364

13 1 -0.228900 2.155945 -4.235004

14 6 1.415661 1.752850 -3.073029

15 6 2.786519 1.547302 -2.899320

16 1 3.440721 1.512802 -3.760682

17 6 3.316542 1.362908 -1.614075

18 1 4.374973 1.182456 -1.493725

19 6 2.478793 1.367013 -0.492923

20 6 1.119287 1.667755 -0.677645

21 1 0.460399 1.700753 0.187616

22 6 0.605869 1.862074 -1.938553

23 6 0.280948 -1.366164 -8.559025

24 1 0.353176 -1.487041 -9.660040

25 1 -0.540115 -1.998479 -8.214814

26 6 1.566470 -1.907108 -7.917600

27 1 1.534894 -2.999399 -7.971005

28 1 2.449807 -1.582846 -8.474444

29 7 1.769923 -1.520711 -6.540688

30 1 2.225630 -0.629145 -6.358360

31 6 0.934460 -2.006951 -5.567431

32 8 0.000000 -2.779824 -5.817104

33 7 1.271647 -1.600570 -4.309364

34 1 1.981553 -0.879739 -4.235004

35 6 0.810182 -2.102424 -3.073029

36 6 -0.053257 -3.186847 -2.899320

37 1 -0.410236 -3.736153 -3.760682

38 6 -0.477958 -3.553663 -1.614075

39 1 -1.163450 -4.380065 -1.493725

40 6 -0.055528 -2.830205 -0.492923

41 6 0.884675 -1.803208 -0.677645

42 1 1.242696 -1.249094 0.187616

43 6 1.309669 -1.455734 -1.938553

44 6 -1.323606 0.439774 -8.559025

45 1 -1.464403 0.437662 -9.660040

46 1 -1.460676 1.466993 -8.214814

47 6 -2.434839 -0.403048 -7.917600

48 1 -3.365003 0.170442 -7.971005

49 1 -2.595688 -1.330172 -8.474444

50 7 -2.201936 -0.772443 -6.540688

51 1 -1.657670 -1.612880 -6.358360

52 6 -2.205301 0.194209 -5.567431

53 8 -2.407398 1.389912 -5.817104

54 7 -2.021958 -0.300994 -4.309364

55 1 -1.752653 -1.276205 -4.235004

56 6 -2.225844 0.349573 -3.073029

57 6 -2.733262 1.639546 -2.899320

58 1 -3.030485 2.223351 -3.760682

59 6 -2.838584 2.190755 -1.614075

60 1 -3.211523 3.197610 -1.493725

61 6 -2.423265 1.463191 -0.492923

62 6 -2.003962 0.135453 -0.677645

63 1 -1.703095 -0.451660 0.187616

64 6 -1.915537 -0.406339 -1.938553

65 1 -1.572169 -1.426066 -2.051855

66 1 -0.448925 2.074571 -2.051855

67 1 2.021094 -0.648505 -2.051855

68 7 -2.300364 1.913070 0.832861

69 1 -1.943007 1.176146 1.457481

70 7 2.806949 1.035639 0.832861

71 1 1.990076 1.094621 1.457481

72 7 -0.506585 -2.948708 0.832861

73 1 -0.047069 -2.270767 1.457481

74 6 -2.408334 3.144953 1.398240

75 6 3.927776 0.513202 1.398240

76 6 -1.519442 -3.658155 1.398240

77 16 -3.272209 4.429767 0.744397

78 16 -2.200186 -5.048700 0.744397

79 16 5.472395 0.618933 0.744397

80 7 3.589358 -0.113436 2.570726

81 1 2.591527 -0.386023 2.627553

82 7 -1.696441 3.165194 2.570726

83 1 -0.961458 2.437340 2.627553

84 7 -1.892918 -3.051757 2.570726

85 1 -1.630069 -2.051317 2.627553

86 6 -2.635381 -3.476166 3.663794

87 6 -2.723929 -2.539903 4.717143

88 6 -3.264803 -4.723860 3.801400

89 6 -3.431032 -2.837674 5.864149

90 1 -2.208871 -1.586173 4.618924

91 6 -3.968066 -5.013420 4.961054

92 1 -3.207608 -5.444444 2.999696

93 6 -4.066036 -4.081889 5.998651

94 1 -4.455734 -5.977584 5.064260

95 6 -1.692758 4.020390 3.663794

96 6 -0.837656 3.628944 4.717143

97 6 -2.458581 5.189332 3.801400

98 6 -0.741982 4.390198 5.864149

99 1 -0.269231 2.706025 4.618924

100 6 -2.357716 5.943156 4.961054

101 1 -3.111223 5.500092 2.999696

102 6 -1.502002 5.562235 5.998651

103 1 -0.083080 4.078990 6.667601

104 1 -2.948872 6.847571 5.064260

105 6 4.328139 -0.544224 3.663794

106 6 3.561585 -1.089040 4.717143

107 6 5.723384 -0.465472 3.801400

108 6 4.173014 -1.552524 5.864149

109 1 2.478102 -1.119852 4.618924

110 6 6.325782 -0.929736 4.961054

111 1 6.318831 -0.055648 2.999696

112 6 5.568037 -1.480346 5.998651

113 1 3.574049 -1.967546 6.667601

114 1 7.404607 -0.869987 5.064260

115 6 -1.408115 6.365663 7.183689

116 7 -1.338363 7.024145 8.135303

117 6 6.216884 -1.963368 7.183689

118 7 6.752269 -2.353016 8.135303

119 16 0.000000 0.000000 3.055155

120 8 0.961932 -1.032311 2.501321

121 8 -1.374973 -0.316902 2.501321

122 8 0.413041 1.349213 2.501321

123 1 -3.490969 -2.111444 6.667601

124 6 -4.808769 -4.402295 7.183689

125 7 -5.413906 -4.671129 8.135303

126 8 0.000000 0.000000 4.526250

---------------------------------------------------------------------

**Table S3.** Cartesian coordinates (in Å) of the urea-bound 1:1 complex [**L**(SO_4_)]^2‒^ calculated at the M06-2X/6-31G(d,p) level of theory in gas phase.

Total energy: -4966.46980076 au.

---------------------------------------------------------------------

Center Atomic Coordinates (Angstroms)

No. Number X Y Z

------------------------------------------------------------

1 7 0.171361 -0.012206 4.234262

2 6 -1.141827 0.505932 4.580480

3 1 -1.141116 0.995717 5.574045

4 1 -1.855093 -0.322983 4.632456

5 6 -1.666830 1.478047 3.520899

6 1 -2.610961 1.908369 3.869030

7 1 -0.954042 2.295676 3.361830

8 7 -1.892066 0.831408 2.247268

9 1 -1.083941 0.677331 1.632267

10 6 -2.929997 -0.043410 2.140642

11 8 -3.781785 -0.186566 3.019794

12 7 -2.935673 -0.738731 0.939951

13 1 -2.210890 -0.519252 0.240914

14 6 -3.836013 -1.716929 0.554305

15 6 -4.951743 -2.126325 1.306573

16 1 -5.144430 -1.651626 2.257099

17 6 -5.791821 -3.132527 0.842109

18 1 -6.641864 -3.437021 1.438426

19 6 -5.545206 -3.741853 -0.385377

20 6 -4.439126 -3.336743 -1.140098

21 1 -4.237469 -3.809668 -2.099832

22 6 -3.595457 -2.342886 -0.686588

23 6 0.394228 -1.382913 4.662765

24 1 -0.045393 -1.578125 5.659928

25 1 1.470262 -1.563018 4.744143

26 6 -0.146446 -2.393229 3.648487

27 1 -0.001914 -3.404874 4.041962

28 1 -1.219210 -2.239119 3.486467

29 7 0.508024 -2.287877 2.363167

30 1 0.169447 -1.573380 1.707931

31 6 1.821832 -2.632153 2.269110

32 8 2.441059 -3.184923 3.180612

33 7 2.379924 -2.309421 1.041076

34 1 1.767462 -1.913342 0.312557

35 6 3.702006 -2.472292 0.663506

36 6 4.709162 -3.024673 1.474698

37 1 4.453118 -3.365443 2.467023

38 6 6.016442 -3.127227 1.011808

39 1 6.779130 -3.548999 1.653434

40 6 6.343009 -2.695078 -0.270787

41 6 5.345642 -2.145273 -1.082488

42 1 5.593588 -1.800303 -2.084813

43 6 4.046044 -2.032030 -0.631272

44 6 1.261416 0.886007 4.578965

45 1 1.680460 0.658145 5.577846

46 1 0.887102 1.913103 4.615369

47 6 2.373573 0.854808 3.527753

48 1 3.196954 1.496162 3.859198

49 1 2.761009 -0.163751 3.406509

50 7 1.915774 1.308192 2.232150

51 1 1.432313 0.631033 1.630324

52 6 1.559897 2.615624 2.091610

53 8 1.765965 3.466179 2.960244

54 7 0.954672 2.885551 0.874344

55 1 0.898234 2.129008 0.174862

56 6 0.397738 4.088886 0.475832

57 6 0.424746 5.275306 1.231814

58 1 0.919846 5.273380 2.191380

59 6 -0.185508 6.428762 0.754447

60 1 -0.166162 7.332958 1.351047

61 6 -0.816244 6.429673 -0.485740

62 6 -0.844414 5.256344 -1.243778

63 1 -1.340322 5.249979 -2.212303

64 6 -0.249300 4.100296 -0.776636

65 1 -0.267763 3.180791 -1.356520

66 1 -2.733665 -2.024790 -1.267943

67 1 3.267258 -1.600102 -1.255184

68 7 -1.534150 7.552608 -0.994166

69 1 -2.317691 7.301961 -1.585346

70 7 -6.299677 -4.829485 -0.910508

71 1 -5.795260 -5.350522 -1.618221

72 7 7.664085 -2.693534 -0.803575

73 1 7.778062 -2.043486 -1.572314

74 6 -1.252117 8.860649 -0.923825

75 6 -7.570072 -5.207130 -0.723066

76 6 8.720766 -3.477150 -0.555040

77 16 0.145396 9.578666 -0.357234

78 16 8.785697 -4.864357 0.373917

79 16 -8.786705 -4.393589 0.082107

80 7 -7.893616 -6.413934 -1.353204

81 1 -8.894186 -6.536663 -1.423912

82 7 -2.248683 9.684091 -1.453441

83 1 -1.904421 10.618815 -1.623094

84 7 9.882518 -3.084563 -1.227980

85 1 10.572506 -3.822955 -1.231619

86 6 10.326845 -1.821228 -1.601320

87 6 11.415491 -1.758875 -2.490333

88 6 9.788847 -0.620468 -1.104332

89 6 11.950598 -0.544795 -2.873360

90 1 11.828101 -2.683849 -2.881461

91 6 10.321740 0.596255 -1.501670

92 1 8.978006 -0.637971 -0.386001

93 6 11.403206 0.649552 -2.387023

94 1 9.904251 1.517419 -1.109684

95 6 -3.625405 9.503878 -1.527917

96 6 -4.345048 10.367204 -2.373700

97 6 -4.332270 8.553773 -0.769480

98 6 -5.721016 10.286951 -2.464735

99 1 -3.801477 11.097712 -2.964963

100 6 -5.712009 8.468740 -0.873889

101 1 -3.808087 7.905021 -0.077985

102 6 -6.420183 9.329682 -1.718756

103 1 -6.266010 10.957547 -3.120460

104 1 -6.250005 7.735343 -0.283001

105 6 -7.110699 -7.532547 -1.613608

106 6 -5.863335 -7.773135 -1.009615

107 6 -7.632891 -8.496008 -2.495991

108 6 -5.164563 -8.935339 -1.297596

109 1 -5.455239 -7.069581 -0.293902

110 6 -6.938121 -9.657863 -2.770341

111 1 -8.592058 -8.310677 -2.969666

112 6 -5.689871 -9.887569 -2.177459

113 1 -4.205407 -9.114391 -0.823872

114 1 -7.350554 -10.392417 -3.453637

115 6 -7.850182 9.251133 -1.817815

116 7 -9.004166 9.205199 -1.903917

117 6 -4.972199 -11.096571 -2.466716

118 7 -4.408661 -12.079933 -2.704656

119 16 0.165465 -0.179555 -0.903189

120 8 0.149500 -0.145595 0.636051

121 8 0.656303 1.152734 -1.364314

122 8 -1.233778 -0.435911 -1.350444

123 8 1.088720 -1.279754 -1.305552

124 6 11.963288 1.908061 -2.790966

125 7 12.432018 2.913546 -3.123518

126 1 0 12.790589 -0.509563 -3.558735

---------------------------------------------------------------------

**Table S4.** Cartesian coordinates (in Å) of [**L**(SO_4_)_2_]^4‒^ calculated at the M06-2X/6-31G(d,p) level of theory in gas phase.

Total energy: -5665.27638350 au.

---------------------------------------------------------------------

Center Atomic Coordinates (Angstroms)

No. Number X Y Z

-----------------------------------------------------------

1 7 0.000000 0.000000 8.027848

2 6 1.077246 -0.926214 8.322978

3 1 1.288096 -0.991947 9.415464

4 1 1.979040 -0.538696 7.837384

5 6 0.853276 -2.361803 7.815239

6 1 1.801992 -2.906119 7.891460

7 1 0.141742 -2.873063 8.486117

8 7 0.384500 -2.401465 6.452871

9 1 0.112242 -1.478622 6.039063

10 6 0.899356 -3.303639 5.545337

11 8 1.795152 -4.108773 5.795475

12 7 0.217222 -3.243191 4.341233

13 1 -0.516924 -2.529990 4.307350

14 6 0.746655 -3.410452 3.046880

15 6 2.081616 -3.682768 2.738497

16 1 2.771044 -3.915400 3.541562

17 6 2.538099 -3.542805 1.426053

18 1 3.587469 -3.684428 1.193398

19 6 1.661078 -3.150792 0.415404

20 6 0.306081 -2.992176 0.708115

21 1 -0.374130 -2.682689 -0.081636

22 6 -0.148861 -3.144952 2.001560

23 6 0.263502 1.396030 8.322978

24 1 0.215003 1.611498 9.415464

25 1 -0.522995 1.983247 7.837384

26 6 1.618744 1.919860 7.815239

27 1 1.615777 3.013631 7.891460

28 1 2.417275 1.559284 8.486117

29 7 1.887480 1.533719 6.452871

30 1 1.224404 0.836515 6.039063

31 6 2.411357 2.430684 5.545337

32 8 2.660726 3.609033 5.795475

33 7 2.700075 1.809715 4.341233

34 1 2.449497 0.817326 4.307350

35 6 2.580210 2.351848 3.046880

36 6 2.148563 3.644116 2.738497

37 1 2.005313 4.357494 3.541562

38 6 1.799110 3.969460 1.426053

39 1 1.397074 4.949053 1.193398

40 6 1.898126 3.013932 0.415404

41 6 2.438260 1.761161 0.708115

42 1 2.510342 1.017339 -0.081636

43 6 2.798039 1.443559 2.001560

44 6 -1.340748 -0.469816 8.322978

45 1 -1.503099 -0.619551 9.415464

46 1 -1.456045 -1.444551 7.837384

47 6 -2.472019 0.441943 7.815239

48 1 -3.417769 -0.107511 7.891460

49 1 -2.559017 1.313780 8.486117

50 7 -2.271980 0.867746 6.452871

51 1 -1.336645 0.642107 6.039063

52 6 -3.310713 0.872954 5.545337

53 8 -4.455877 0.499739 5.795475

54 7 -2.917296 1.433476 4.341233

55 1 -1.932574 1.712664 4.307350

56 6 -3.326865 1.058603 3.046880

57 6 -4.230179 0.038652 2.738497

58 1 -4.776358 -0.442095 3.541562

59 6 -4.337209 -0.426655 1.426053

60 1 -4.984542 -1.264625 1.193398

61 6 -3.559205 0.136860 0.415404

62 6 -2.744341 1.231014 0.708115

63 1 -2.136212 1.665350 -0.081636

64 6 -2.649178 1.701393 2.001560

65 1 -1.939526 2.484797 2.235316

66 1 -1.182135 -2.922077 2.235316

67 1 3.121660 0.437280 2.235316

68 7 -3.383179 -0.415897 -0.885166

69 1 -2.403826 -0.406164 -1.192132

70 7 2.051767 -2.721971 -0.885166

71 1 1.553662 -1.878692 -1.192132

72 7 1.331413 3.137868 -0.885166

73 1 0.850164 2.284856 -1.192132

74 6 -4.270670 -0.837063 -1.797829

75 6 2.860252 -3.279977 -1.797829

76 6 1.410417 4.117040 -1.797829

77 16 -5.946379 -0.864497 -1.559308

78 16 2.224514 5.581964 -1.559308

79 16 3.721866 -4.717467 -1.559308

80 7 2.853898 -2.522580 -2.953994

81 1 2.236199 -1.682225 -2.900074

82 7 -3.611567 -1.210258 -2.953994

83 1 -2.574949 -1.095493 -2.900074

84 7 0.757670 3.732838 -2.953994

85 1 0.338750 2.777718 -2.900074

86 6 0.574342 4.331849 -4.177654

87 6 0.053872 3.482959 -5.187072

88 6 0.826066 5.682960 -4.490012

89 6 -0.201093 3.968459 -6.451941

90 1 -0.124880 2.432649 -4.952111

91 6 0.565970 6.154995 -5.765067

92 1 1.222558 6.334493 -3.724982

93 6 0.052386 5.316285 -6.762753

94 1 0.758836 7.198377 -5.998015

95 6 -4.038663 -1.668529 -4.177654

96 6 -3.043267 -1.694825 -5.187072

97 6 -5.334621 -2.126086 -4.490012

98 6 -3.336240 -2.158381 -6.451941

99 1 -2.044296 -1.324474 -4.952111

100 6 -5.613367 -2.587353 -5.765067

101 1 -6.097110 -2.108480 -3.724982

102 6 -4.630231 -2.612775 -6.762753

103 1 -2.563738 -2.166281 -7.213991

104 1 -6.613396 -2.942017 -5.998015

105 6 3.464320 -2.663320 -4.177654

106 6 2.989395 -1.788134 -5.187072

107 6 4.508555 -3.556874 -4.490012

108 6 3.537333 -1.810078 -6.451941

109 1 2.169176 -1.108175 -4.952111

110 6 5.047397 -3.567642 -5.765067

111 1 4.874553 -4.226012 -3.724982

112 6 4.577845 -2.703510 -6.762753

113 1 3.157924 -1.137122 -7.213991

114 1 5.854559 -4.256360 -5.998015

115 6 -4.954905 -3.094070 -8.071608

116 7 -5.238639 -3.488101 -9.126550

117 6 5.156996 -2.744038 -8.071608

118 7 5.640104 -2.792744 -9.126550

119 16 0.000000 0.000000 -2.960632

120 8 -0.409995 1.353830 -2.432151

121 8 -0.967453 -1.031981 -2.432151

122 8 1.377449 -0.321849 -2.432151

123 8 0.000000 0.000000 -4.447954

124 1 -0.594185 3.303403 -7.213991

125 6 -0.202091 5.838108 -8.071608

126 7 -0.401465 6.280845 -9.126550

127 16 0.000000 0.000000 3.775795

128 8 -1.126931 -0.881210 3.378035

129 8 0.000000 0.000000 5.332643

130 8 1.326616 -0.535345 3.378035

131 8 -0.199685 1.416556 3.378035

---------------------------------------------------------------------
